# IFN-signaling gene expression as a diagnostic biomarker for monogenic interferonopathies

**DOI:** 10.1172/jci.insight.178456

**Published:** 2024-06-17

**Authors:** Laura A. Adang, Russell D’Aiello, Asako Takanohashi, Sarah Woidill, Francesco Gavazzi, Edward M. Behrens, Kathleen E. Sullivan, Raphaela Goldbach-Mansky, Adriana A. de Jesus, AGS Clinical Trial Readiness Workgroup, Adeline Vanderver, Justine Shults

**Affiliations:** 1 Division of Neurology, Department of Pediatrics, Children’s Hospital of Philadelphia (CHOP), Philadelphia, Pennsylvania, USA.; 2 Department of Neurology, Perelman School of Medicine, University of Pennsylvania, Philadelphia, Pennsylvania, USA.; 3 Department of Biomedical and Health Informatics,; 4 Division of Rheumatology, Department of Pediatrics, and; 5 Department of Allergy Immunology, Department of Pediatrics, CHOP, Philadelphia, Pennsylvania, USA.; 6 Translational Autoinflammatory Diseases Section, National Institute of Allergy and Infectious Diseases, NIH, Bethesda, Maryland, USA.; 7 AGS Clinical Trial Readiness Workgroup details are available in Supplemental Acknowledgments.; 8 Department of Biostatistics, Epidemiology, and Informatics, Perelman School of Medicine at the University of Pennsylvania, Pennsylvania, USA.; 9 Department of Pediatrics, CHOP, Philadelphia, Pennsylvania, USA.

**Keywords:** Inflammation, Neuroscience, Innate immunity, Monogenic diseases, Neurological disorders

## Abstract

IFN-signaling gene (ISG) expression scores are potential markers of inflammation with significance from cancer to genetic syndromes. In Aicardi Goutières Syndrome (AGS), a disorder of abnormal DNA and RNA metabolism, this score has potential as a diagnostic biomarker, although the approach to ISG calculation has not been standardized or validated. To optimize ISG calculation and validate ISG as a diagnostic biomarker, mRNA levels of 36 type I IFN response genes were quantified from 997 samples (including 334 AGS), and samples were randomized into training and test data sets. An independent validation cohort (*n* = 122) was also collected. ISGs were calculated using all potential combinations up to 6 genes. A 4-gene approach (*IFI44L*, *IFI27*, *USP18*, *IFI6*) was the best-performing model (AUC of 0.8872 [training data set], 0.9245 [test data set]). The majority of top-performing gene combinations included *IFI44L*. Performance of *IFI44L* alone was 0.8762 (training data set) and 0.9580 (test data set) by AUC. The top approaches were able to discriminate individuals with genetic interferonopathy from control samples. This study validates the context of use for the ISG score as a diagnostic biomarker and underscores the importance of *IFI44L* in diagnosis of genetic interferonopathies.

## Abstract 2

Interferon signaling gene expression scores are validated as a diagnostic biomarker for type I interferonopathies, including Aicardi Goutières Syndrome.

## Graphical abstract

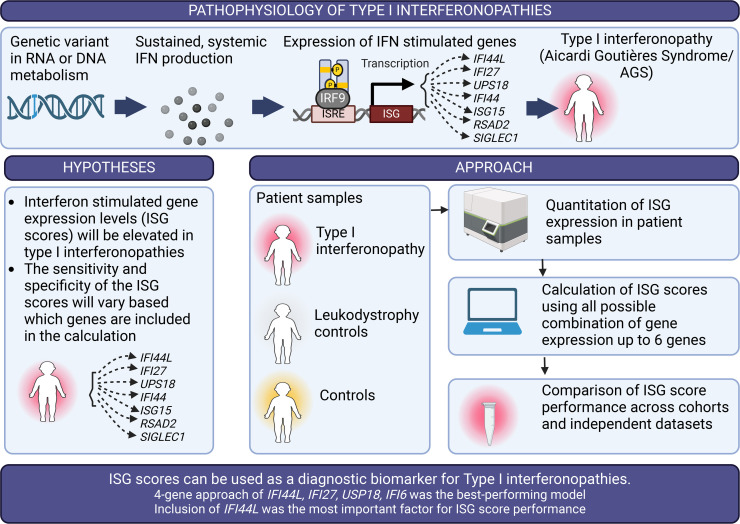

## Introduction

Genetic interferonopathies are a diverse group of conditions characterized by systemic inflammation with variable clinical manifestations. The most common of this family of disorders is Aicardi Goutières Syndrome (AGS) (1–4), although there are other disorders including Familial Chilblain Lupus (FCL), STING-associated vasculopathy with onset in infancy (SAVI), and chronic atypical neutrophilic dermatosis with lipodystrophy and elevated temperatures (CANDLE) (5). Activation of the IFN pathway can also occur with acquired rheumatologic disorders, including systemic lupus erythematous (SLE) and dermatomyositis (6, 7).

**Figure 1:**
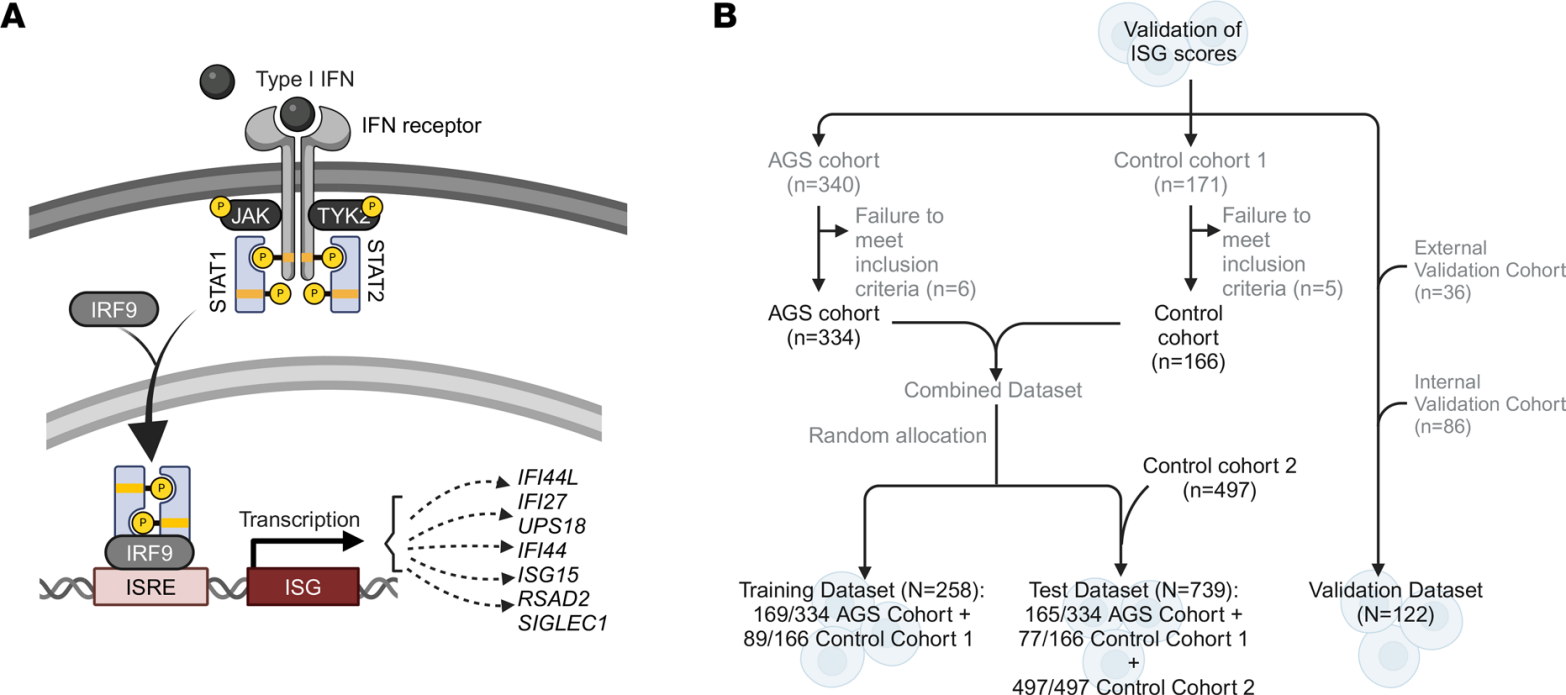
Biomarker validation in AGS. (**A**) Schematic depicting the IFN signaling pathway, which is constitutively activated in Aicardi Goutières Syndrome and other genetic interferonopathies. Stimulation at the IFN receptor results in expression of IFN signaling genes via the JAK/STAT pathway. The STAT/IRF9 complex binds to IFN-stimulated response elements (ISRE), leading to the transcription of IFN-signaling genes (ISG). The mRNA levels of these ISGs can be quantitated and combined as an “IFN-stimulated gene expression score,” a surrogate for IFN pathway activity. Various mathematical approaches have been used to calculate this score. AGS, Aicardi Goutières Syndrome; JAK, janus kinase; JAKi, JAK inhibitor; STAT, signal transducer and activator of transcription; ISRE, IFN-stimulated response elements. Made with BioRender. (**B**) Concert Diagram for sample ISG testing and validation.

Direct measurement of type I IFNs as diagnostic or prognostic biomarkers has been limited by methodologic challenges with accurate quantification of IFNs. Historically, cell toxicity assays were required to capture IFN activity (8), although multiplex digital ELISA approaches are a potential direction (9). To date, researchers have relied on surrogate markers of IFN activity, particularly the measurement of mRNA levels of IFN-responsive genes (IFN signaling gene expression signatures) as an indirect measure of IFN activity (1, 10, 11). Initial reports have used individual gene expression levels by quantitative PCR (qPCR) to calculate disease-specific scores (10–13). More recently, groups have used larger multiplexed RNA counting approaches (nanoString nCounter assay) (14–16). This has led to a proliferation of nonstandardized IFN-signaling gene (ISG) expression signaling scores. ISG scores reflect a calculation of mRNA levels of known ISGs normalized to the expression of housekeeping genes (Figure 1A). The 2 most common approaches include the levels of *IFI27*, *IFI44*, *IFI44L*, *ISG15*, and *RSAD2* with either *UPS18* (referred to as NIH-6 in this manuscript) or *SIGLEC1* (referred to as AGS-6) (1, 10, 11, 14, 16, 17). While ISG scores have been used as exploratory pharmacoresponsive biomarkers in clinical trials (17–19), the role of the ISG score in diagnosis and disease stratification is poorly defined (20–22). Challenges with utilizing the ISG score include the heterogeneity in gene expression levels compared with the severity of the clinical phenotype. Additionally, in AGS, a subset of patients with *RNASEH2B*-related disease with severe clinical AGS has been reported to have normal ISG scores (10), suggesting that there may be genetic heterogeneity in ISG scores.

In this report, we characterize the pattern of type I ISG expression using a multiplex digital gene expression measurement approach (nanoString nCounter Analysis System) (23). This cohort includes individuals with genetic interferonopathies compared with other known leukodystrophies, healthy controls, and hospitalized controls. We compare common existing ISG score calculations to alternative approaches to optimize the identification of individuals with genetic interferonopathy compared with nonaffected controls and to maximize performance in the *RNASEH2B* subcohort.

## Results

### Model development.

In total, 1,119 samples were tested across 2 institutions, the CHOP and the NIH (Figure 1B). In brief, mRNA levels of IFN-associated genes were measured and normalized to housekeeping genes (Figure 1A). These mRNA levels were used for comparison across patient cohorts using different ISG calculation approaches.

In the model development phase, the expression of IFN response gene from 997 samples was measured, and samples were randomized into training (*n* = 258) and test (*n* = 739) data sets (Figure 1B). The randomized division into training and validation cohorts was completed at the patient level to avoid samples from the same patient in both data sets. The training data set was used for model building, and the validation data set was used to compare the ability of approaches to correctly classify AGS verses non-AGS samples. The complete data sets for the model development phase (training and test data sets) includes 334 samples from individuals with a molecular diagnosis of AGS. Two groups of controls were also used. Control Cohort 1 includes 166 samples with non-AGS leukodystrophies (e.g., *POLR3*-related leukodystrophy, Pelizaeus Merzbacher disease, *TUBB4A*-associated leukodystrophies, and Alexander disease). Control Cohort 2 includes 497 samples from individuals with identified non-AGS medical conditions collected during clinical care. Cohort 2 was from a set of discarded clinical samples that were completely anonymized prior to inclusion. Detailed clinical information about treatment status or length of diseases in this cohort is not available. The Control Cohort 2 was included only as part of the test cohort to evaluate specificity in a real-world context with non-AGS inflammatory conditions in which ISGs may be elevated.

In the validation phase, a second independent validation cohort (*n* = 122; including 36 samples from the NIH) was used. The CHOP validation data set included samples from individuals with genetic interferonopathies (*n* = 65) and noninflammatory leukodystrophies (*n* = 21). The NIH cohort included samples from patients affected by AGS (*n* = 6), CANDLE (*n* = 18), and SAVI (*n* = 12).

### Generation of ISG scores.

The expression of individual genes as well as all 2-, 3-, 4-, 5-, and 6-gene combinations were compared within the Test Data set (*n* = 739; Table 1). The AUC (with 95% CI) was obtained for the ROC curve for each classifier in the training and test data sets and compared by AUC (Supplemental Table 4; supplemental material available online with this article; https://doi.org/10.1172/jci.insight.178456DS1). The validation data set, using the best cut-off points based on predicted probabilities from models established in the training, was used to estimate sensitivity, specificity, and the number needed to misdiagnose a patient (Supplemental Tables 5 and 6) for tests based on the best cut-off values in the validation data set. The threshold for positive-negative for each ISG approach was determined to maximize sensitivity and sensitivity (Supplemental Table 13).

**Figure 2:**
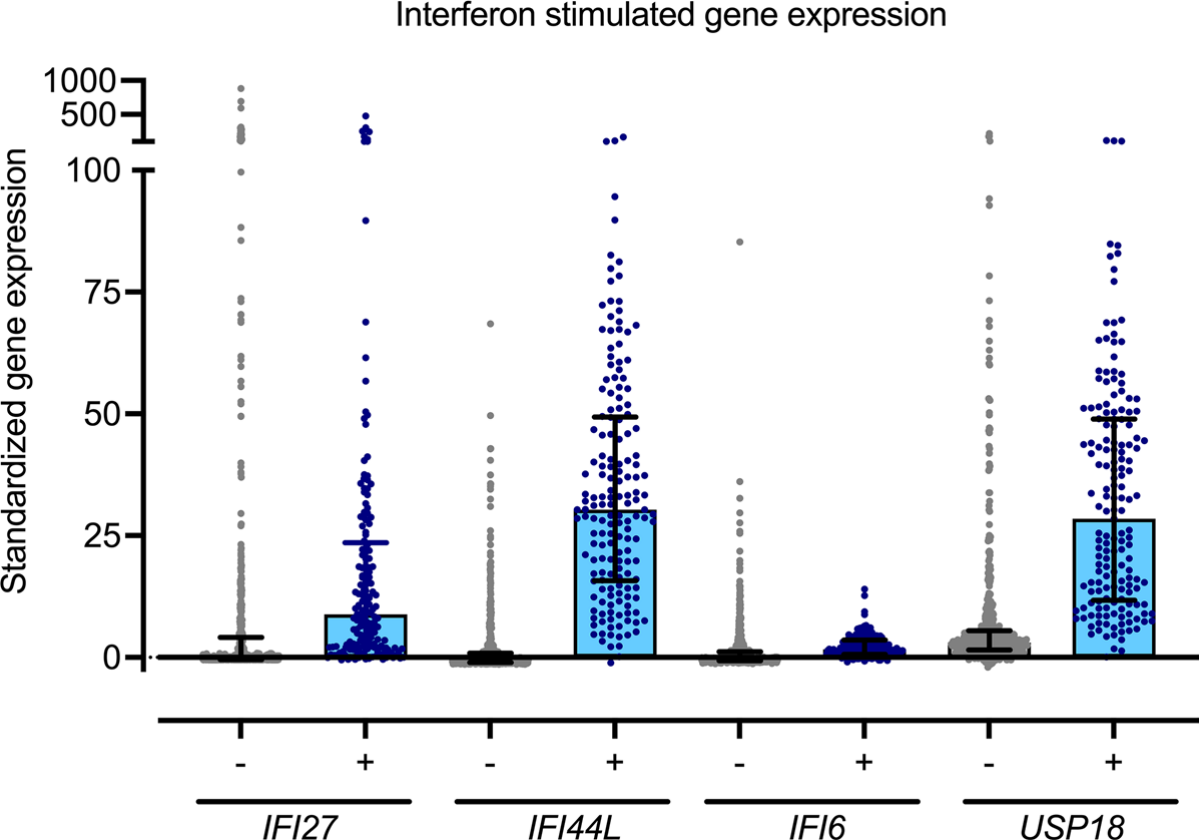
The performance of the top AUC performers was compared in the test data set. Standardized gene expression scores for *IFI27*, *IFI44L*, *IFI6*, and *USP18* were compared across 2 populations: the non-AGS control cohorts (–) (*n* = 574) and the AGS cohorts (+) (*n* = 165). The control cohorts include patients with noninflammatory leukodystrophies as well as samples collected during hospitalization or out-patient care on nonleukodystrophy patients.

Overall, the single gene best able to distinguish between individuals with AGS from those without AGS (determined by maximal AUC) was *IFI44L*, which was included in the top-performing combinations (Table 1). The top-performing combination in the training data set was a 4-gene median approach including *IFI44L*, *IFI27*, *IFI6*, and *USP18* (AUC, 0.8872). The standardized gene expression scores for the top-performing single genes were compared between non-AGS control cohorts (*n* = 574) and the AGS cohorts (*n* = 165) (Figure 2). The expression of these genes was higher in the AGS population compared with controls (Table 2).

**Figure 3:**
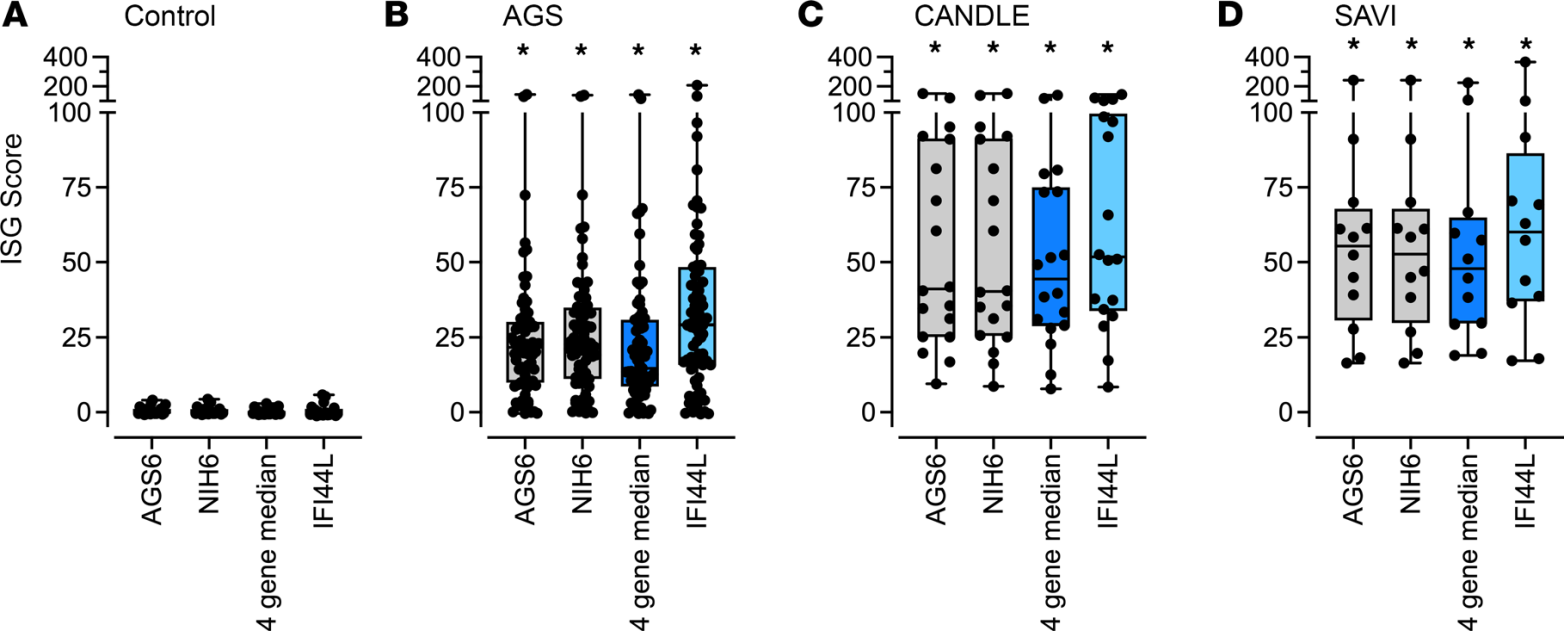
Comparison of performance between 4 ISG calculation approaches in the validation data set. The performance of the AGS-6, NIH-6, 4-gene median, and IFI44L alone were compared across 4 populations. (**A**–**D**) This included a control cohort (**A**) and 3 interferonopathies: AGS (**B**), CANDLE (**C**), and SAVI (**D**). Boxes represent median with 25th to 75th percentiles, with whiskers spanning minimum to maximum values. The calculated ISG scores for control samples were compared with the values from the AGS, CANDLE, and SAVI cohorts by Kruskal-Wallis test with Dunn’s correction for multiple comparisons. *P_adj_ < 0.0001.

For further comparisons of performance, we compared the performance of 2 common scoring approaches AGS-6 (1, 10, 11) and NIH-6 (14) with the alternative top-performing approach (4-gene median) and *IFI44L* alone in the validation phase. The AGS-6 and NIH-6 share common genes of *IFI27*, *IFI44*, *IFI44L*, *ISG15*, and *RSAD2*, with the addition of either *SIGLEC1* (AGS-6) or *UPS18* (NIH-6) (Table 1 and Figure 3, A–D). This cohort included control samples and 3 interferonopathies: AGS, CANDLE, and SAVI (Supplemental Tables 2 and 3). These approaches were able to discriminate between individuals with interferonopathy and without interferonopathy (Figure 3 and Supplemental Table 9). Of note, the IFN score was higher in individuals with CANDLE and SAVI compared with those with AGS (Figure 3, C and D).

**Figure 4:**
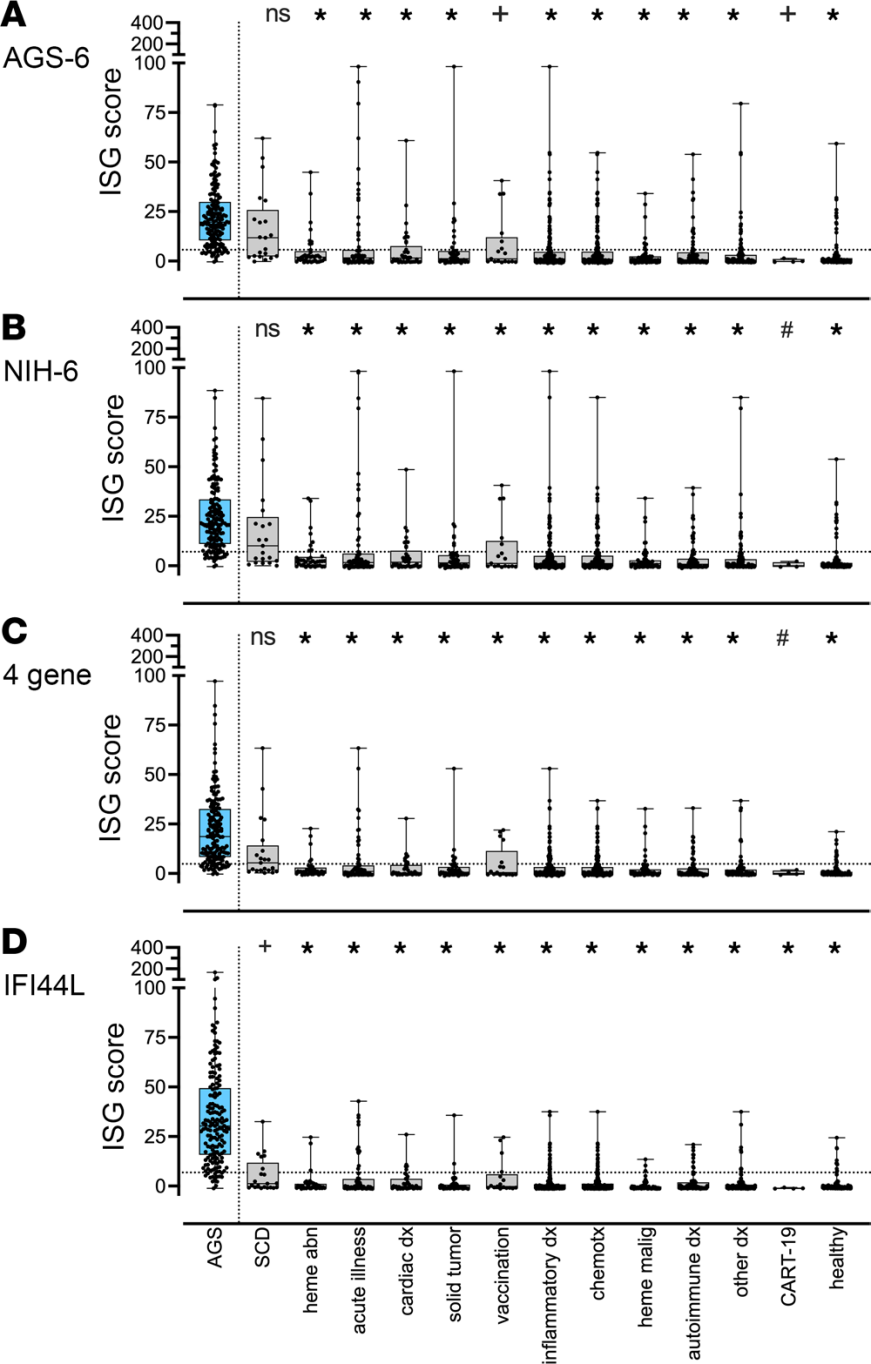
Comparison of performance of 4 ISG calculation approaches by disease state in the test data set. From 382 patients, ISG were calculated from 547 samples. Non-AGS controls were categorized by disease state and may be represented more than once. Categories included: sickle cell disease (SCD), hematologic abnormalities (heme abn, e.g., primary anemia, primary thrombocytopenia), acute illness (e.g., any sample collected during a period with concern for active infection), cardiac disease, solid tumor, vaccination (administration immediately preceding sample collection), inflammatory disease (e.g., chronic infection, FPIES, celiac disease), chemotherapy (concurrent with sample collection), heme (hematologic) malignancy, autoimmune disease (e.g. systemic lupus erythromatosis, rheumatoid arthritis), other (includes any other organ-based diagnosis requiring specialist follow up, e.g. epilepsy, liver disease), and CART-19 administration. Boxes represent median with 25th to 75th percentiles, with whiskers spanning minimum to maximum values. The calculated ISG scores for AGS samples were compared with the values from control cohorts by Kruskal-Wallis test with Dunn’s correction for multiple comparisons. *P_adj_ < 0.0001, ^+^*P*_adj_ ≤ 0.001, ^#^*P*_adj_ ≤ 0.0055.

In a real-world setting, the AGS diagnosis status may be unknown at the time of testing, and other acquired inflammatory states may be present. To address this challenge, we next compared the performance of 4 ISG calculation approaches by disease state in the test data set (382 patients, 547 samples) (Figure 4). Controls included samples obtained from clinical care of both hospitalized and out-patient visits. Disease state was categorized as sickle cell disease (SCD), hematologic abnormalities (e.g., primary anemia, primary thrombocytopenia), acute illness (e.g., any sample collected during active infection), cardiac disease, solid tumor, vaccination (administration immediately preceding sample collection), inflammatory disease (e.g., chronic infection, FPIES, celiac disease), chemotherapy (concurrent with sample collection), hematologic malignancy, autoimmune disease (e.g., SLE, rheumatoid arthritis), other (includes any other organ-based diagnosis requiring specialist follow up, e.g., epilepsy, liver disease), and CART-19 administration (Supplemental Table 1). SCD was notable for increased ISG scores as calculated across all approaches.

**Figure 5:**
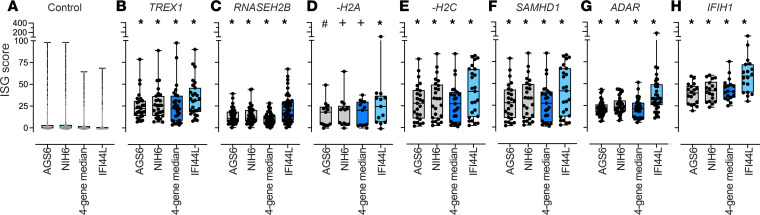
Comparison of performance of 4 ISG calculation approaches by genotype in the test data set. (**A**–**H**) The performance of the control, AGS-6, NIH-6, 4-gene median, and IFI44L alone were compared across the AGS genotypes. There was a total of 739 samples from 611 unique patients. Boxes represent median with 25th to 75th percentiles, with whiskers spanning minimum to maximum values. The calculated ISG scores for AGS samples were compared with the values from controls by Kruskal-Wallis test with Dunn’s correction for multiple comparisons. *P_adj_ < 0.0001, ^+^*P*_adj_ ≤ 0.0009, ^#^*P*_adj_ = 0.0018.

**Figure 6:**
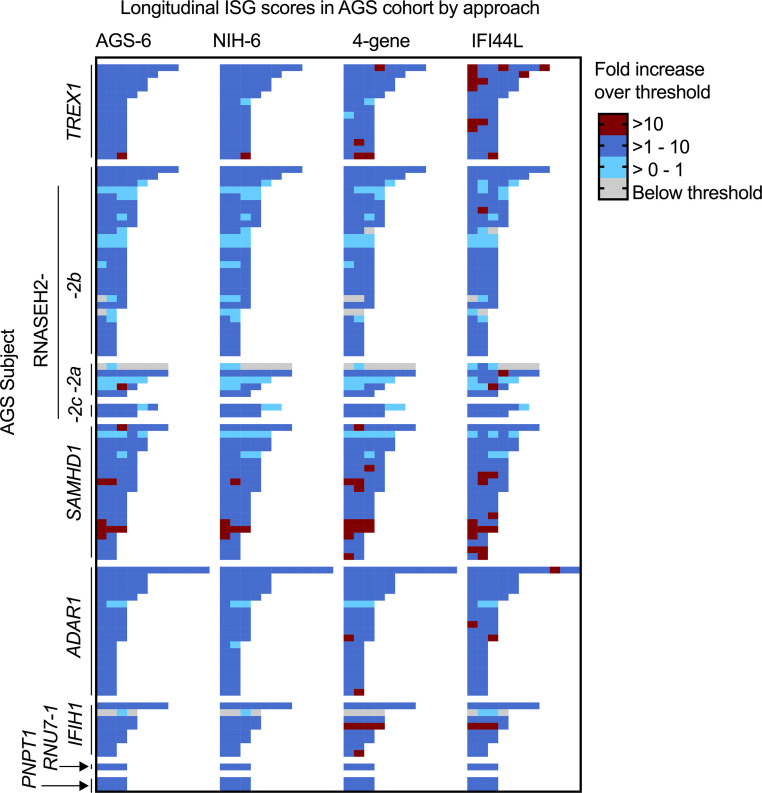
Patient-level change in the ISG score. Across 81 patients with AGS, 301 total samples were collected, and ISG scores were calculated by 4 approaches: AGS-6, NIH-6, 4-gene median, and IFI44L single gene expression. The fold change over approach-specific threshold is shown in the heatmap. Patient have between 1 and 10 additional samples after the initial sample.

Next, the performance of 4 ISG calculation approaches was compared by AGS genotype in the test data set (Figure 5). Six known genotypes were represented. The patients with *RNASEH2B-* and *RNASEH2A*-related AGS were noted to have lower IFN activity as measured by all 4 approaches (Figure 5 and Table 2). Additionally, samples were collected longitudinally from 99 patients with AGS across all data sets (9 genotypes, *n* = 358 total samples) (Figure 6). These samples were collected from patients without janus kinase inhibitor or other immunomodulatory treatment (18). Variability of ISG performance across the samples from a patient was noted across all approaches, although most samples were elevated consistently above the threshold for normal.

## Discussion

ISG scores have evolved as an important surrogate measure of type I IFN activity and have been used in clinical trials to measure disease activity (18). Since ISG measurements are introduced into clinical settings, it is essential to understand their role as a biomarker in AGS. In this study, we evaluated the role of ISG scores as a diagnostic biomarker in AGS and other monogenic interferonopathies. Furthermore, since ISG scores can be calculated using a range of approaches, we evaluated the performance of each method for discriminating between individuals affected by AGS, monogenic interferonopathies, and other disorders, including systemic illnesses.

Based on our analyses, most calculation approaches for ISG scores were capable of distinguishing AGS and other interferonopathies from unaffected controls. The most important variable was the inclusion of *IFI44L*. IFN-induced protein 44-like (IFI44L) plays a role in the immune response to viral infections and has been implicated in the pathogenesis of SLE (24, 25). Interestingly, *IFI44L* expression negatively modulates the proinflammatory state induced by IFN treatment or infection (26, 27). Because *IFI44L* was sufficient to distinguish AGS from hospitalized controls (often with concurrent infection), we hypothesize that the chronic inflammation associated with genetic interferonopathies may lead to a preferential upregulation of *IFI44L*. While ISG scores represent a single value, scores that include multiple genes in the calculation are a representation of expression of both positive and negative regulators of expression. It is also possible that different inflammatory states, such as AGS versus CANDLE versus SAVI versus infection, may have characteristic patterns of ISG expression. Larger cohorts of less-frequent interferonopathies will be needed to explore this question.

These approaches underscore the flexibility of ISG calculations. While the 4-gene median was the best-performing approach, the differences were small among the top performers. It is also possible that, in the future, new additions to IFN multiplexed gene expression panels may result in improved predictive performance. Over time, an iterative approach to model development may be helpful to exploit newly available data.

Finally, samples from patients with *RNASEH2B*-related AGS have been previously reported to have lower IFN activity, irrespective of severity (10, 28). Our data suggest that the estimated probability of correct classification is significantly lower for *RNASEH2*-related AGS for all classification methods tested. As previously reported (16, 18), there can be variability in ISG levels at the patient level, underscoring the value of repeated sample collection for the diagnosis of AGS. We hypothesize that patients with nongenetically driven increased IFN activity, such as in the context of an acute infection, will not have sustained elevation of ISG scores.

There are potential clinical benefits to the formal validation of ISG scores as a diagnostic biomarker, specifically as this biomarker could identify children with AGS who would be eligible for therapeutic intervention. This is important as treatments become available in AGS, as ISG scores may be available within a few days, whereas genetic testing may take weeks or longer (in the case of noncoding or other variants not easily accessible by standard testing approaches).

There are inherent limitations to the use of ISG scores as a diagnostic biomarker. Importantly, the finding that a subset of patients with RNASEH2-related disease do not have elevated ISG scores limits the diagnostic power of this approach in this subset of patients; in this context, treatment should not be withheld if patients with RNASEH2-related disease do not have an elevated score. This will also be important in patient selection for future clinical trials. Conversely, since ISG scores can be elevated in a number of conditions, there is a risk for using ISG scores in isolation. To reduce this risk of misdiagnosis, ISGs should be repeated and used in combination with genetic and clinical information to reach the diagnosis of AGS. Finally, we acknowledge that the inability to account for intrapatient association of measurements due to being unable to group ISG measurements at the patient level in the anonymized control data set was an inherent limitation.

ISG scores have additional potential for other biomarker roles. This includes as a susceptibility or risk biomarker, identifying presymptomatic individuals prior to disease onset (10, 11, 15, 17, 29). This may support correct patient stratification as newborn screening is developed. ISG scores have also been used effectively as pharmacodynamic biomarkers in the context of clinical trials (17, 18). Future research will be necessary to fully understand the role of ISG scores in the care of individuals affected by monogenic interferonopathies.

This work defines the potential for ISG scores as a biomarker in AGS. AGS is a serious condition with no validated diagnostic biomarkers. Therefore, there is an urgent need for biomarkers for patient selection for imminent clinical trials. According to Biomarkers, EndpointS, and other Tools (BEST) criteria, ISG scores are a diagnostic biomarker, capable of identifying individuals with monogenic interferonopathies. There is a strong biologic rationale for the use of ISG scores in AGS, since it is a measurement of the downstream IFN receptor activation and since scores are stably elevated in individuals with AGS. This report also outlines the analytical performance of ISG scores in the AGS population. The source, RNA extracted from patient blood has established methodology for ISG calculation, and criteria for interpretation can reliably differentiate between monogenic interferonopathies and other conditions.

## Methods

*Sex as a biological variable*. Age and sex has not been shown to affect ISGs; thus, they were not considered as biologic variables in analyses (14). Information on patient sex and age at collection of samples as available is provided in Supplemental Tables 2 and 3.

### Cohort identification and clinical characterization

Patients with leukodystrophies or interferonopathies were consented under IRB protocols at the NIH and CHOP (14-011236 CHOP). Hospital control samples were collected under an IRB-exemption (21-019194).

Disease samples and controls were collected under a leukodystrophy-associated biorepository protocol, the Myelin Disorders Biorepository and Natural History Study, approved by CHOP IRB (IRB 14-011236). Individuals were identified as having either AGS (Disease Cohort) based on molecular identification of 1 of the AGS-related genes (*TREX1*, *RNASEH2A RNASEH2B*, *RNASEH2C*, *SAMHD1*, *ADAR1*, *IFIH1*, *RNU7-1*, *PNPT1*, with no patients with *LSM11*) or as having a non–AGS-related leukodystrophy (Control Cohort 1) (Supplemental Table 1). An additional cohort of children with similar age distribution is evaluated under an IRB exemption from the pathology lab at CHOP and includes children with identified nonexclusive medical categories as varied as recent vaccinations, acute systemic illness, solid tumor, hematologic malignancy, SCD, and other hematologic abnormalities such as anemia, chemotherapy regimens, cardiac disease, autoimmune conditions, inflammatory disease, and other organ-based disease (Control Cohort 2; Supplemental Table 1). Categories included SCD, hematologic abnormalities (e.g., primary anemia, primary thrombocytopenia), acute illness (e.g., any sample collected during a period with concern for active infection), cardiac disease (e.g., dilated cardiomyopathy and other structural defects), solid tumor, vaccination (administration immediately preceding sample collection), inflammatory disease (e.g., chronic infection, solid organ transplant, and tumors), chemotherapy (concurrent with sample collection), hematologic malignancy, autoimmune disease (e.g., systemic lupus erythromatosis, rheumatoid arthritis), other (includes any other organ-based diagnosis requiring specialist follow up, e.g., epilepsy, liver disease), and CART-19 administration (Supplemental Table 1). A subset of these children has had no identifiable medical disorder and had blood drawn during well-child visits. Samples have been anonymized after collection, and no further clinical information is available for these samples.

### Sample collection

In the Disease Cohort and Control Cohort 1, samples have been drawn during a venipuncture immediately into PAXgene RNA blood tubes (BD Biosciences). In Control Cohort 2, samples have been drawn into EDTA tubes and used for clinical purposes. Residual samples are kept at room temperature for 24 hours and at 4°C for another 24 hours, before being transferred to a PAXgene RNA blood tube. All samples collected into PAXgene RNA blood tubes and extracted RNA are stored at –80°C until processing for gene expression measures. After the RNA extraction using PAXgene RNA extraction kit (PreAnalytiX), an RNA integrity number (RIN) above 8, as measured by TapeStation (Agilent), is required for processing via nanoString.

### ISG expression measures

NanoString nCounter assay (nanoString Technologies) approaches have been previously described for the measurement of ISG expression (14). This approach uses complementary DNA synthetic oligos to provide direct measurement of gene expression counts without reverse transcription or amplification (Supplemental Table 12). The Elements system requires Capture (probe A) and tag (probe B) probe DNA oligos were designed by nanoString and synthesized by Integrated DNA Technologies (IDT). Probe A and B each contain the gene-specific sequences; probe A also has a gene-specific sequence that binds the reporter tag, whereas probe B binds to a universal capture tag. Nucleotide sequences for probes A and B are listed in Supplemental Tables 10 and 11, respectively. After hybridization at 65°C for 16 hours, RNA counts were immediately obtained by using nCounter Sprint Profiler (nanoString) or FLEX Prep Station/Digital Analyzer (nanoString) at “High Sensitivity” mode with maximum Fields of View of 256 and 555, respectively. Data were processed with nSolver software (nanoString), which included assessment of quality of the runs. Data were then exported to Excel (Microsoft Corporation), and the raw RNA counts were used to create *Z* scores using Stata 18 (Stata Corp.).

### Generation of calibration standards

Synthetic DNA oligonucleotides of each of the 36 ISG target genes included in the score and the 4 housekeeping genes were designed by nanoString and synthesized by IDT (Supplemental Table 12). These synthetic DNA oligonucleotides were used as a calibration standard to check run and reagent lot consistency.

### Statistics

#### Creation of training, validation analytic data sets.

First, 2 analytic data sets were generated to create distinct cohorts (Figure 1B and Supplemental Tables 2 and 3). One of the analytic data sets contains confirmed AGS (Disease Cohort) and non-AGS samples (Control Cohort 1); this was randomly split at the patient level (when patient IDs were available), into a training and validation data set. The second analytic data set (Control Cohort 2) only contains non-AGS samples and was reserved for evaluation of specificity (the probability that a non-AGS sample is classified as non-AGS).

#### Characteristics of the data sets.

The following number of unique individuals is included in each data set: training, *n* = 119; test, *n* = 611; and validation, *n* = 83. Information on the sex and age distribution of cohort 2 and the normative control data set was limited as per the IRB study exempt approval.

#### Creation of Z-scores on which all analyses are based.

In order to calculate *Z* scores from each set of gene expression counts, normalization approaches are applied. First, we standardize the genes expression counts by multiplying by a conversion factor based on the geometric mean (gmean) of the housekeeping genes (HK) *(ALAS1*, *HPRT1*, *TBP*, and *TUBB*) (ConvFactor), where ConvFactor = 1000/gmean_HK and gmean_HK = the geometric mean of the 4 housekeeping genes. For each standardized gene, we then generate a gene specific *Z* score, where *Z* score = (gene count – Cmean_gene)/Csd_gene) where Cmean_gene and Csd_gene are the mean and SD of that gene count from a standard data set, respectively, of 47 control individuals with non-AGS leukodystrophies or family members without evidence of ongoing illness as previously described (15, 18, 30–32).

#### ISG score comparisons.

*Z*-scores by group are visually displayed with box plots. Patient-level changes in ISG score are evaluated with a heatmap. The ISG scores between cohorts were compared by Kruskal-Wallis test with Dunn’s correction for multiple comparisons, resulting in an adjusted *P* value (Figure 3, Figure 4, and Figure 5).

#### Evaluation of the performance of the classification variables.

Accuracy was estimated as the percentage of samples that were correctly classified out of the total cohorts. The potential classifiers for AGS (described in Supplemental Table 4 and Supplemental Table 13) were compared by first constructing receiver operator characteristic (ROC) curves, which display the estimated sensitivity versus 1-specificity for tests that consider each observed value of the classifier as a cut-point (AGS is predicted if ≥ the cut-point). Empirical (nonparametric) ROC curves were obtained using the roccurve command in Stata 18 AUC (33). The AUC was obtained in the training and validation data sets (with 95% CI) and was compared with the AUC for the median of NIH 6 genes (reference approach for our analyses, with previously published cut-point of 1.97) in the validation data set, with the test implemented in the comproc command in Stata 18 (33). To account for correlation within samples from the same patient, the 95% CI for AUCs were estimated using bootstrap resampling at the patient level (for samples with available patient identification variable). Higher values of the AUC are suggestive of better performance because higher values occur when the estimated sensitivity and specificity values are larger across all observed values of a classifier.

#### Calculation of optimal cut-point by ISG calculation approach.

In addition to estimated AUC, which is a summary measure across all potential cut-points for a classifier, we obtained optimal cut-points on the ROC curves using several criteria (34). See Supplemental Table 13 for a description of the criteria for selection of optimal cut-points. The threshold for each best-performing approach was, therefore, determined by the point on the ROC curve at which Youden’s index (sensitivity + specificity – 1) was maximal (35).

#### Sensitivity and specificity.

Next, we estimated the sensitivity and specificity of tests that classify a sample as AGS if the classifier is ≥ the cut-point (Supplemental Tables 5 and 6). Sensitivity was estimated as the percentage (with 95% CI) of AGS samples with a classifier value ≥ the cut-point — i.e., sensitivity was estimated as the percentage of AGS samples that are correctly classified. Specificity was estimated as the percentage (with 95% CI) of non-AGS samples with a classifier value < the cut-point — i.e., specificity was estimated as the percentage of non-AGS samples that are correctly classified. We estimated both sensitivity and specificity (with 95% CI) in the training and validation data sets. In the non-AGS data set (Control Cohort 2), we only estimated specificity (with 95% CI) because no AGS samples were available in this data set. To account for correlation within samples from the same patient, logistic generalized estimating equation (GEE) models for the binary classifier were fit with no covariates (only a constant) and an exchangeable correlation structure that assumes equal correlation between any 2 samples on the same patient. The estimated correlation from this model was then used to adjust the 95% CI for sensitivity or specificity, using the prtest command in Stata 18; on a few occasions, the GEE estimates fail to converge due to estimated correlations > 1. If this happens, the assumed correlation is set to 0.99.

#### Positive and negative predictive value.

Positive predictive values (PPV) and negative predictive values (NPV) are provided in Supplemental Tables 7 and 8 that correspond to the respective sensitivity (*sens*) and specificity (*spec*) values in Supplemental Tables 5 and 6. PPV and NPV values are provided for assumed prevalence (*prev*) of AGS = 5%, 10%, and 15%.

The PPV value = 
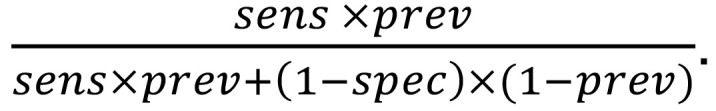


The NPV value = 
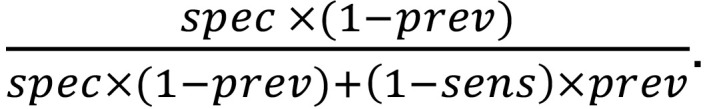


#### Number needed to misdiagnose.

When we identified the best cut point with respect to the number needed to misdiagnose (Supplemental Tables 5 and 6), we assumed the *prev* of AGS was 5%, 10%, and 15% and that the harm of a false-negative result (*C* to *C*) was twice that of a false-positive result (Supplemental Tables 7 and 8). With these assumptions, we estimated the number of samples that will need to be tested before a sample is misdiagnosed using the following formula that is also provided in Supplemental Tables 5 and 6: 1/(*C* to *C* × *prev* × [1 – *sens*] + [1 – *prev*] × [1 – *spec*]).

#### Genotype and correct classification.

To determine if the likelihood of correct classification varied according to genotype, we fit the first-order Markov conditional linear expectation approach (MARK1ML) with a logit link function for the outcome of correct classification in AGS validation samples by approach. This classifies a sample as AGS if the estimated probability of AGS was ≥ the Youden cut-point of 0.65, and the Youden AGS-6 gene classifier classifies a sample as AGS if the median of the AGS-6 genes was ≥ the Youden cut-point of 5.69. The MARKLML logistic model includes an indicator variable for *RNASEH2* genotype (versus other genotypes). To account for the correlation of measurements within the repeated measurements on a patient, we assume a first-order autoregressive AR(1) correlation structure, for which the correlation between adjacent measurements on a patient is α.

#### Genotype and longitudinal stability.

The MARK1ML model also involves a model for the conditional expectation, which can be used to evaluate longitudinal stability of classification. According to Equation 2 in ref. 36, the probability of correct classification given the prior observation on a patient is correctly classified is


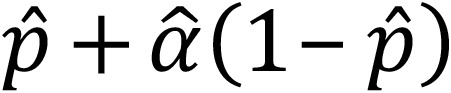


where 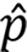 is the estimated probability of correct classification and 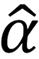 is the estimated correlation parameter.

This approach provides an estimated conditional probability for *RNASEH2-*related genotypes and other genotypes (combined).

#### Genotype and cross-sectional agreement.

We obtained the percentage of AGS samples in the validation data set by ability to correctly classify by approach. We then obtained the percentage of samples for which there was disagreement between approaches for the *RNASEH2*-related samples and non–*RNASEH2*-related samples. We then compared the odds of disagreement between *RNASEH2* related and non–*RNASEH2-*related samples by fitting a logistic model of disagreement on an indicator variable for *RNASEH2*-related samples (versus non–*RNASEH2-*related samples), with adjusted standard errors to account for clustering within patients.

### Study approval

Written informed consent was received prior to participation, with the exception of the hospital control cohort, which was collected under an IRB-exemption excluding the need for consent (IRB 21-019194). Patients with leukodystrophies or interferonopathies were consented under IRB protocols at the NIH and CHOP (14-011236 CHOP). Disease samples and controls were collected under a leukodystrophy-associated biorepository protocol, the Myelin Disorders Biorepository and Natural History Study, approved by CHOP IRB (IRB 14-011236).

### Data availability

Anonymized data not published as part of this article will be made available by request from qualified investigators. Additional control cohort data are not available as per the IRB-exemption. Values for all data points in graphs are reported in the Supporting Data Values file.

## Author contributions

Authorship order of first authors was assigned by experimental design contribution and manuscript development. LAA contributed the primary experimental design, figure generation, writing of the manuscript, and critical review of manuscript. RD contributed the primary statistical support, experimental design, supported writing, and critical review of the manuscript. AT contributed the primary assay development and execution, supported writing, and critical review of the manuscript. Other authors. SW contributed conducting experiments, writing the manuscript, and critical review of manuscript. FG contributed writing the manuscript and critical review of manuscript. EMB contributed writing the manuscript, critical review of manuscript, and control data set generation. KES contributed writing the manuscript, critical review of manuscript, and control data set generation. RGM provided external validation and contributed writing the manuscript and critical review of manuscript. AADJ provided external validation and contributed writing the manuscript and critical review of manuscript. AV contributed the funding source, writing the manuscript, critical review of manuscript, design and analysis, and final review of all data. JS contributed statistical methodology design and analysis, writing the manuscript, critical review of manuscript, and final review of all data. The AGS Clinical Trial Readiness Workgroup recruited patients, provided samples, and reviewed the overall contribution of ISGs to biomarker and clinical trial readiness in AGS.

## Supplementary Material

Supplemental data

Supporting data values

## Figures and Tables

**Table 1:**
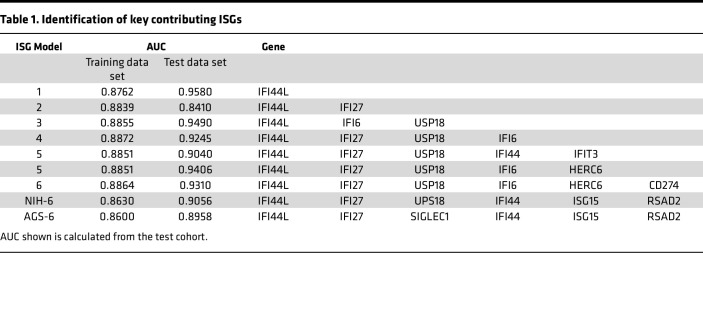
Identification of key contributing ISGs

**Table 2:**
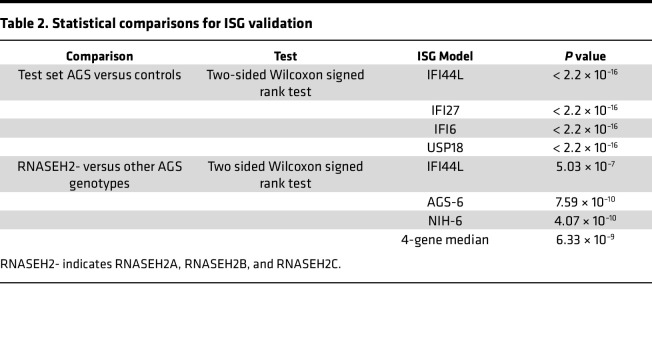
Statistical comparisons for ISG validation

## Version 1 06/17/2024

In-Press Preview

## Version 2 07/22/2024

Electronic publication
