# Supplementary material for: IFN-signaling gene expression as a diagnostic biomarker for monogenic interferonopathies
Source: JCI Insight. 2024 Jun 17;9(14):e178456. doi: 10.1172/jci.insight.178456 (PMC11383167; doi:10.1172/jci.insight.178456)
Supplement: Supplemental data [file jciinsight-9-178456-s218.pdf]

Supplemental Materials

Supplemental Acknowledgement

AGS Clinical Trial Readiness Workgroup Members:

Simona Orcesi, Department of Brain and Behavioral Sciences, University of Pavia, Pavia, Italy, Child Neurology and Psychiatry Unit, IRCCS Mondino Foundation, Pavia, Italy. Elisa Fazzi, Department of Clinical and Experimental Sciences, Azienda Socio Sanitaria Territoriale Spedali Civili, Brescia, Italy. Stephanie Keller, Department of Neurology, Children's Healthcare of Atlanta, Atlanta, Georgia, and Department of Pediatrics and Neurology, Emory University School of Medicine, Atlanta, Georgia. Jamie Fraser, Division of Genetics and Metabolism, Children's National Medical Center, Washington, DC. James Krueger, Laboratory of Investigative Dermatology, The Rockefeller University, New York, New York. Amit Bar-Or, Department of Neurology, Perelman School of Medicine at the University of Pennsylvania, Philadelphia, Pennsylvania. James Treat, Dermatology Section, Children's Hospital of Philadelphia, Philadelphia, Pennsylvania. Allan Glanzman, Department of Physical Therapy, Children's Hospital of Philadelphia, Philadelphia, Pennsylvania. Scott Lorch, Division of Neonatology, Children's Hospital of Philadelphia, Philadelphia, Pennsylvania, and Department of Pediatrics, Perelman School of Medicine at the University of Pennsylvania, Philadelphia, Pennsylvania. Sara DeMauro, Division of Neonatology, Children's Hospital of Philadelphia, Philadelphia, Pennsylvania. Shanice Beerepoot, Department of Child Neurology, VU University Medical Center, Amsterdam, Netherlands.

Conflicts of interest:

LA receives research support for research from Eli Lilly, Takeda, Illumina, Boehringer Ingelhiem, Biogen, Orchard Therapeutics. LA serves on the scientific advisory boards of the MLD Foundation, CureMLD, and Don't Forget Morgan Foundation. She is also a consultant for Takeda, Biogen, and Orchard Therapeutics. RD receives grant support from Takeda, Biogen, Orchard Therapeutics, Affinia, and Sana. EMB received research support from AB2Bio and is a consultant for Sobi and Genzyme. RGM and AADJ receive research support through the NIH intramural research program (IRP) at NIAID. AV receives grant and in-kind support for research from Eli Lilly, Gilead, Takeda, Illumina, Boehringer Ingelhiem, Biogen, Homology, Ionis, Passage Bio, Affinia, Sana, Sanofi, Myrtelle, and Orchard Therapeutics. She also serves on the scientific advisory boards of the European Leukodystrophy Association and the United Leukodystrophy Foundation as well as in an unpaid capacity for Takeda, Ionis, Biogen, and Illumina.

Supplemental Table 1. Examples of health conditions present in Control Cohorts

Cohort	Control Cohort 1	Control Cohort 2
**Health Conditions**	4H syndrome, Alexander disease, Angelman syndrome, Cockayne disease, Metachromatic Leukodystrophy, Multiple Sulfatase deficiency, Mitochondrial disease, Pelizaeus Merzbacher disease and Pelizaeus Merzbacher like disease, TUBB4A associated leukodystrophy, X-linked Adrenoleukodystrophy, Vanishing White Matter disease, and other primary genetic disorders with a confirmed mutation unrelated to AGS	Developmental disorder, medication requiring blood monitoring including for acne or epilepsy, anemia and blood cell dyscrasia, well visit with or without vaccination, endocrinologic evaluation, inpatient care for trauma, celiac disease, ulcerative colitis, Crohn’s disease, Lupus, Juvenile dermatomyositis, Sickle cell disease, renal failure, structural heart disease, hematologic malignancy or solid tumor, acute viral or bacterial illness.

Supplemental Table 2. Comparison of cohorts by sample

	Train (N=258)	Test (N=739)	Internal validation (N=86)	External validation (N=36)	Overall (N=1119)
**Sex**					
Female	98 (38.0%)	347 (47.0%)	38 (44.2%)	18 (50.0%)	501 (44.8%)
Male	159 (61.6%)	387 (52.4%)	48 (55.8%)	18 (50.0%)	612 (54.7%)
Missing	1 (0.4%)	5 (0.7%)	0 (0%)	0 (0%)	6 (0.5%)
**Age at Sample (years)**					
Mean (SD)	7.71 (8.05)	6.93 (6.35)	12.1 (15.0)	11.6 (9.39)	7.67 (7.98)
Median [Min, Max]	4.33 [0.142, 57.1]	5.00 [0.0100, 44.8]	7.40 [0.444, 70.2]	7.49 [2.29, 40.8]	5.00 [0.0100, 70.2]
Missing	2 (0.8%)	3 (0.4%)	0 (0%)	0 (0%)	5 (0.4%)
**Interferonopathy**					
Yes	169 (65.5%)	165 (22.3%)	65 (75.6%)	36 (100%)	435 (38.9%)
No	89 (34.5%)	574 (77.7%)	21 (24.4%)	0 (0%)	684 (61.1%)
**Genotype**					
ADAR1	27 (10.5%)	35 (4.7%)	14 (16.3%)	0 (0%)	76 (6.8%)
IFIH1	16 (6.2%)	18 (2.4%)	2 (2.3%)	0 (0%)	36 (3.2%)
PNPT1	0 (0%)	0 (0%)	6 (7.0%)	0 (0%)	6 (0.5%)
RNASEH2A	11 (4.3%)	13 (1.8%)	4 (4.7%)	0 (0%)	28 (2.5%)
RNASEH2B	42 (16.3%)	47 (6.4%)	16 (18.6%)	0 (0%)	105 (9.4%)
RNASEH2C	10 (3.9%)	0 (0%)	1 (1.2%)	0 (0%)	11 (1.0%)
RNU7-1	0 (0%)	0 (0%)	4 (4.7%)	0 (0%)	4 (0.4%)
SAMHD1	36 (14.0%)	25 (3.4%)	12 (14.0%)	0 (0%)	73 (6.5%)
TREX1	27 (10.5%)	27 (3.7%)	6 (7.0%)	0 (0%)	60 (5.4%)
CANDLE	0 (0%)	0 (0%)	0 (0%)	0 (0%)	0 (0%)
SAVI	0 (0%)	0 (0%)	0 (0%)	0 (0%)	0 (0%)
Non-AGS cohort	89 (34.5%)	574 (77.7%)	21 (24.4%)	36 (100%)	720 (64.3%)

Supplemental Table 3. Comparison of cohorts by subject

	Train (N=119)	Test (N=611)	Internal validation (N=59)	External validation (N=24)	Overall (N=813)
**Sex**					
Female	52 (43.7%)	272 (44.5%)	24 (40.7%)	11 (45.8%)	359 (44.2%)
Male	66 (55.5%)	334 (54.7%)	35 (59.3%)	13 (54.2%)	448 (55.1%)
Missing	1 (0.8%)	5 (0.8%)	0 (0%)	0 (0%)	6 (0.7%)
**Interferonopathy**					
No	62 (52.1%)	554 (90.7%)	18 (30.5%)	0 (0%)	634 (78.0%)
Yes	57 (47.9%)	57 (9.3%)	41 (69.5%)	24 (100%)	179 (22.0%)
**Genotype**					
ADAR1	11 (9.2%)	11 (1.8%)	10 (16.9%)	0 (0%)	32 (3.9%)
IFIH1	6 (5.0%)	7 (1.1%)	2 (3.4%)	0 (0%)	15 (1.8%)
PNPT1	0 (0%)	0 (0%)	2 (3.4%)	0 (0%)	2 (0.2%)
RNASEH2A	3 (2.5%)	4 (0.7%)	2 (3.4%)	0 (0%)	9 (1.1%)
RNASEH2B	15 (12.6%)	17 (2.8%)	12 (20.3%)	0 (0%)	44 (5.4%)
RNASEH2C	2 (1.7%)	0 (0%)	1 (1.7%)	0 (0%)	3 (0.4%)
RNU7-1	0 (0%)	0 (0%)	2 (3.4%)	0 (0%)	2 (0.2%)
SAMHD1	13 (10.9%)	8 (1.3%)	6 (10.2%)	0 (0%)	27 (3.3%)
TREX1	7 (5.9%)	10 (1.6%)	4 (6.8%)	0 (0%)	21 (2.6%)
CANDLE	0 (0%)	0 (0%)	0 (0%)	0 (0%)	0 (0%)
SAVI	0 (0%)	0 (0%)	0 (0%)	0 (0%)	0 (0%)
Non-AGS cohort	62 (52.1%)	554 (90.7%)	18 (30.5%)	24 (100%)	658 (80.9%)

Supplemental Table 4: Comparison of different approaches to AGS 6 genes by AUC (with 95% CI) and p-value (for comparison with median AGS 6 genes) in Training Data Set (N = 258) and Test Data Set (N = 739). (See Consort Diagram in Figure 1b.)

	**Training Data Set (N = 258)**		**Test Data Set (N = 739)**	
**Approach**	**AUC (95% CI)**	**p-value for comparison with median AGS 6**	**AUC (95% CI)**	**p-value for comparison with median AGS 6**
Median of AGS 6 genes	.860 (95% CI = .783 to .928)	NA (Reference approach)	.896 (95% CI = .868 to .921)	NA (Reference approach)
Median of NIH 6 genes	.863 (95% CI = .797 to .927)	0.716	.906 (95% CI = .877 to .931)	<0.0005
4-gene median	.887 (95% CI = .816 to .945)	0.023	.925 (95% CI = .894 to .950)	<0.0005
IFI44L	.876 (95% CI = .801 to .943)	0.151	.958 (95% CI = .938 to .975)	<0.0005

Supplemental Table 5: Sensitivity, Specificity, and Number of samples needed to misdiagnose (NNM) for a test based on the optimal cut-point (obtained using Youden Index) in the Test Data Set that contains the AGS Cohort (N = 165) and Control Cohort 1 (N = 77). (See Consort Diagram in Figure 1b.)

**Variable**	**Optimal cut-point for Classification of non-AGS (< cut-off) [Youden Index]**	**Specificity = [#<cut-off]/[#non-AGS samples] (% and 95% CI)**	**Sensitivity = [#>=cut-off]/[#AGS Samples] (% and 95% CI)***	**Number needed to misdiagnose (NNM) for prevalence (prev) = 10% (NNM for prev = 5%, NNM for prev = 15%)****
Median of NIH 6 genes	7.05	67/77 (87.01%; 95% CI = 77.64 to 96.38)	141/165 (85.45%; 95% CI = 75.76 to 95.15)	6.9 (7.3, 6.5)
Median of AGS 6 genes	5.69	67/77 (87.01%; 95% CI = 77.64 to 96.38)	145/165 (87.88%; 95% CI = 79.02 to 96.74)	7.1 (7.4, 6.8)
4-gene median	4.82	68/77 (88.31%; 95% CI = 79.18 to 97.44)	141/165 (85.45%; 95% CI = 76.23 to 94.67)	7.4 (8.0, 7.0)
IFI44L	6.82	67/77 (87.01%; 95% CI = 77.64 to 96.38)	151/165 (91.52%; 95% CI = 85.51 to 97.52)	7.5 (7.6, 7.4)

*The values in this column are equal in Supplemental Tables 5 and 6.

**Number Samples Needed to Misdiagnose (NNM), assuming prevalence (*prev*) of AGS = 10% (5%, 15%); harm of false negative (*C*) is twice that of false positive results; and sensitivity (*se*), specificity (*sp*) are as estimated for a test that classifies a sample as AGS if the classifier is >= each of the cut-points in Table 3A. The weighted number to misdiagnose (1) (NNM) = $\frac{1}{C \times prev(1-se)+(1-prev)\times(1-sp)}$.

Supplemental Table 6: Sensitivity, Specificity, and Number of samples needed to misdiagnose (NNM) for a test based on the optimal cut-point (obtained using Youden Index) in the Test Data Set that contains the AGS Cohort (N = 165) and Control Cohorts 1 and 2 combined (N = 574). (See Consort Diagram in Figure 1b.)

**Variable:**	**Cut off Value for Classification of non-AGS (< cut-off) [Youden Index]**	**Specificity = [#<cut-off]/[#non-AGS samples] (% and 95% CI)**	**Sensitivity = [#>=cut-off]/[#AGS Samples] (% and 95% CI)***	**Number needed to misdiagnose (NNM) for prevalence (prev) = 10% (NNM for prev = 5%, NNM for prev = 15%)**
Median of NIH 6 genes	7.05	479/574 (83.45%; 95% CI = 80.32 to 86.58)	141/165 (85.45%; 95% CI = 75.76 to 95.15)	5.6 (5.8, 5.4)
Median of AGS 6 genes (2-4)	5.69	466/574 (81.18%; 95% CI = 77.90 to 84.47)	145/165 (87.88%; 95% CI = 79.02 to 96.74)	5.2(5.2,5.1)
4-gene median	4.82	484/574 (84.32%; 95% CI = 81.25 to 87.39)	141/165 (85.45%; 95% CI = 76.23 to 94.67)	5.9 (6.1,5.7)
IFI44L	6.82	508/574 (88.5%; 95% CI = 85.78 to 91.22)	151/165 (91.52%; 95% CI = 85.51 to 97.52)	8.5 (8.3,8.1)

*The values in this column are equal in Supplemental Tables 5 and 6.

**Number Samples Needed to Misdiagnose (NNM), assuming prevalence (*prev*) of AGS = 10%, (5%, 15%); harm of false negative (*C*) is twice that of false positive results; and sensitivity (*se*), specificity (*sp*) are as estimated for a test that classifies a sample as AGS if the classifier is >= each of the cut-points in Table 4A. The weighted number to misdiagnose (NNM) $= \frac{1}{C \times prev(1-se)+(1-prev)\times(1-sp)}$.

Supplemental Table 7: False Positive, False Negative, Positive Predictive Value (PPV), Negative Predictive Value (PPV) for a test based on the optimal cut-point (obtained using Youden Index) in the Test Data Set that contains the AGS Cohort (N = 165) and Control Cohort 1 (N = 77). (See Consort Diagram in Figure 1b.)

Variable	Cut off Value for Classification of non-AGS (< cut-off) [Youden Index]	False Positive = [#>=cut-off]/[#non-AGS samples] (% and 95% CI)	False Negative = [#<cut-off]/[#AGS Samples] (% and 95% CI)*	Positive Predictive Value (PPV) for prevalence (prev) = 10% (PPV for prev = 5%, PPV for prev = 15%)**	Negative Predictive Value (NPV) for prevalence (prev) = 10% (NPV for prev = 5%, NPV for prev = 15%)***
Median of NIH 6 genes	7.05	10/77 (12.99%; 95% CI = 3.62 to 22.36)	24/165 (14.55%; 95% CI = 4.85 to 24.24)	42.23% (25.72,53.73)	98.18% (99.13,97.13)
Median of AGS 6 genes (2-4)	5.69	10/77 (12.99%; 95% CI = 3.62 to 22.36)	20/165 (12.12%; 95% CI = 3.26 to 20.98)	42.92% (26.26,54.42)	98.48% (99.27,97.60)
4-gene median	4.82	9/77 (11.69%; 95% CI = 2.56 to 20.82)	24/165 (14.55%; 95% CI = 9.55 to 20.87)	44.82% (27.79,56.34)	98.20% (99.14,97.18)
IFI44L	6.82	10/77 (12.99%; 95% CI = 3.62 to 22.36)	14/165 (8.48%; 95% CI = 2.48 to 14.49)	43.91% (27.05,55.43)	98.93% (99.49,98.31)

*The values in this column are equal in Supplemental Tables 7 and 8.

**Positive Predictive Value, assuming prevalence (*prev*) of AGS = 10% (5%,15%); sensitivity (*se*), and specificity (*sp*) are as estimated for a test that classifies a sample as AGS if the classifier is >= each of the cut-points in Table 3B. The positive predictive value $= \frac{sens \times prev}{sens \times prev + (1-spec) \times (1-prev)}$.

**Negative Predictive Value, assuming prevalence (*prev*) of AGS = 10% (5%,15%); sensitivity (*se*), and specificity (*sp*) are as estimated for a test that classifies a sample as AGS if the classifier is >= each of the cut-points in Table 3B. The negative predictive value $= \frac{spec \times (1-prev)}{spec \times (1-prev) + (1-sens) \times prev}$.

Supplemental Table 8: False Positive, False Negative, Positive Predictive Value (PPV), Negative Predictive Value (PPV) for a test based on the optimal cut-point (obtained using Youden Index) in the Test Data Set that contains the AGS Cohort (N = 165) and Control Cohorts 1 and 2 combined (N = 574). (See Consort Diagram in Figure 1b.)

Variable: [All variables are predicted probabilities except for median of NIH 6 genes]	Cut off Value for Classification of non-AGS (< cut-off) [Youden Index]	False Positive = [#>=cut-off]/[#non-AGS samples] (% and 95% CI)	False Negative = [#<cut-off]/[#AGS Samples] (% and 95% CI)*	Positive Predictive Value (PPV) for prevalence (prev) = 10% (PPV for prev = 5%, PPV for prev = 15%)**	Negative Predictive Value (NPV) for prevalence (prev) = 10% (NPV for prev = 5%, NPV for prev = 15%)***
Median of NIH 6 genes	7.05	95/574 (16.55%; 95% CI = 13.42 to 19.68)	24/165 (14.55%; 95% CI = 4.85 to 24.24)	PPV = 36.46% (21.37, 47.68)	NPV = 98.10% (99.09, 97.02)
Median of AGS 6 genes (2-4)	5.69	108/574 (18.82%; 95% CI = 15.53 to 22.1)	20/165 (12.12%; 95% CI = 3.26 to 20.98)	PPV = 34.17% (19.73, 45.18)	NPV = 98.37% (99.22, 97.43)
4-gene median	4.82	90/574 (15.68%; 95% CI = 12.61 to 18.75)	24/165 (14.55%; 95% CI = 9.55 to 20.87)	PPV = 37.72% (22.29, 49.03)	NPV = 98.12% (99.10, 97.05)
IFI44L	6.82	66/574 (11.5%; 95% CI = 8.8 to 14.22)	14/165 (8.48%; 95% CI = 2.48 to 14.49)	PPV = 46.93% (29.52, 58.41)	NPV = 98.95% (99.50,98.34)

*The values in this column are equal in Supplemental Tables 7 and 8.

**Positive Predictive Value, assuming prevalence (*prev*) of AGS = 10% (5%,15%); sensitivity (*se*), and specificity (*sp*) are as estimated for a test that classifies a sample as AGS if the classifier is >= each of the cut-points in Table 4B. The positive predictive value $= \frac{sens \times prev}{sens \times prev + (1-spec)\times(1-prev)}$.

**Negative Predictive Value, assuming prevalence (*prev*) of AGS = 10% (5%,15%); sensitivity (*se*), and specificity (*sp*) are as estimated for a test that classifies a sample as AGS if the classifier is >= each of the cut-points in Table 4B. The negative predictive value $= \frac{spec \times (1-prev)}{spec \times (1-prev) + (1-sens)\times prev}$.

Supplemental Table 9. **Comparison of score performance in external validation dataset** containing the external individuals affected by AGS, CANDLE and SAVI.

Cohort	NIH-6 positives	AGS-6 positives	4-gene median positives	*IFI44L* positives
**CANDLE**	100% (n=18)	100% (n=18)	100% (n=18)	100% (n=18)
**External AGS cohort**	100% (n=6)	100% (n=6)	100% (n=6)	100% (n=6)
**SAVI**	100% (n=12)	100% (n=12)	100% (n=12)	100% (n=12)

Supplemental Table 10. Nucleotide sequences for probe A

Gene Name	Accession	Position	Tag	Name	Sequence
BCL2L1	NM_001191.2	261-360	T001	NM_001191.2:260_T001	TGAGTCTCGTCTCTGGTTAGTGATTCTCTTCTAAGATCCAAAGCCAAGATCCTCAAGACCTAAGCGACAGCGTGACCTTGTTTCA
CD274	NM_014143.3	1246-1345	T002	NM_014143.3:1245_T002	GAGCAATGGATGATTTGCTTGGAGGCTCCTTGTTCAGAAGTATCCCATCCTCTTCTTTTCTTGGTGTTGAGAAGATGCTC
CXCL10	NM_001565.1	41-140	T003	NM_001565.1:40_T003	AATCAGAATCGCAGTTTGATTCATGGTGCTGAGACTGGAGGTTCCTCTGCCACAATTCTGCGGGTTAGCAGGAAGGTTAGGGAAC
DDX60	NM_017631.5	661-760	T004	NM_017631.5:660_T004	GGTAACTCTCTTCCAAGAAACTTCCCCACTCTTTTGATAAGCATCTCGAACTGTTGAGATTATTGAGCTTCATCATGACCAGAAG
GBP1	NM_002053.1	2111-2210	T005	NM_002053.1:2110_T005	TTTACCTAAGACTCTGCTCAGTATCCATTTGTCTACTGCTGGTCATCTGGCAAAGACGCCTATCTTCCAGTTTGATCGGGAAACT
HERC5	NM_016323.2	3092-3191	T006	NM_016323.2:3091_T006	ACTGAAACATGTCAGTGCTCTTATAGGGTCTCTTTCATTCCAACTTTCAGCGAACCTAACTCCTCGCTACATTCCTATTGTTTTC
HERC6	NM_001165136.1	2076-2175	T007	NM_001165136.1:2075_T007	CTTTAACCAGGCGACTTCGTCTGACTCTAAGTATAAATCTGGGTGATGGACCAATTTGGTTTTACTCCCCTCGATTATGCGGAGT
IDO1	NM_002164.3	51-150	T008	NM_002164.3:50_T008	CCTCTGGTGCCCCTCAGTGTCTGAAGAGTTTTCAGAGCATCTTATAATAGCTTTCGGGTTATATCTATCATTTACTTGACACCCT
IFI27	NM_005532.3	391-490	T009	NM_005532.3:390_T009	GAGCCCAGGATGAACTTGGTCAATCCGGAGAGTCCAGTTGCCAACAGCCACTTTTTTTCCAAATTTTGCAAGAGCC
IFI44	NM_006417.4	751-850	T010	NM_006417.4:750_T010	CAGTTGTATTAGTGCCCACCAAAGCCTGATGCGTTACATGCCCTTGGAAACACCGTGTGGACGGCAACTCAGAGATAACGCATAT
IFI44L	NM_006820.2	941-1040	T011	NM_006820.2:940_T011	CTTCTGCCCCATCTAGCCCCATAGTGTCACACAACATAAATGGCAGAGATCCTGGAGTTTATGTATTGCCAACGAGTTTGTCTTT
IFI6	NM_002038.3	181-280	T012	NM_002038.3:180_T012	GAGCCGCTGTCCGAGCTCTCCGAGCACTTTTTCTTACCTGCAGATAAGGTTGTTATTGTGGAGGATGTTACTACA
IFIT1	NM_001548.3	1441-1540	T013	NM_001548.3:1440_T013	TGTAGACGAACCCAAGGAGGCTCAAGCTTTCCAGATCTAATGCCTTTCTCCTTCCTTCCTGTGTTCCAGCTACAAACTTAGAAAC
IFIT2	NM_001547.4	1996-2095	T014	NM_001547.4:1995_T014	ATGAACTTAGCACATTACTGGCTATGCAGGACTAACCTCTATGGGATGCACATAAAATTGGTTTTGCCTTTCAGCAATTCAACTT
IFIT3	NM_001031683.2	1471-1570	T015	NM_001031683.2:1470_T015	GCTGACAGGAAAATACTGCCTATGCCTGAAGGGGCATCCCTTAGCAGCTGGTCAAGACTTGCATGAGGACCCGCAAATTCCT
IFIT5	NM_012420.2	3588-3687	T016	NM_012420.2:3587_T016	TTGTTTAAAGTGCAGAAATCTCAGGCCCCTTCTGTGTATGAACACAGCCCCTTTCGTTGGGACGCTTGAAGCGCAAGTAGAAAAC
IFNA2	NM_000605.3	12-111	T017	NM_000605.3:11_T017	GTTGCAGATGCTGCTAGACTGGTTGAAATGGGTGAGCCTAAACCTTAGGCCCAGCAGACCTGCAATATCAAAGTTATAAGCGCGT
ISG15	NM_005101.3	306-405	T018	NM_005101.3:305_T018	CTCAGAGGTTCGTCGCATTTGTCCACCACCAGCAGGACCCTGCCAATGCACTCGATCTTGTCATTTTTTTGCG
LAMP3	NM_014398.3	879-978	T019	NM_014398.3:878_T019	CTGCTTCCGTTTAGAACCTGATAAATTCCAGTCTTGACTGACGATGGCTGCAAACTGGAGAGAGAAGTGAAGACGATTTAACCCA
LY6E	NM_002346.2	381-480	T020	NM_002346.2:380_T020	TGATGCCCATGGAAGCCACACCAACATTGACGCCTTCTGGGATGGGCGATTGCTGCATTCCGCTCAACGCTTGAGGAAGTA
MX1	NM_002462.2	1486-1585	T021	NM_002462.2:1485_T021	CCTACAGTTTCCTCTCCTTGCATGAGAGCAGTGATGTCCTGATTAAAGGCCTGAGGCTGTTAAAGCTGTAGCAACTCTTCCACGA
OAS1	NM_016816.2	6-105	T022	NM_016816.2:5_T022	ATCTCTTCTGCTGACTGAGCTTGGACTGCTGTTGGTTTCGTTTCCTCAGACTAGGACGCAAATCACTTGAAGAAGTGAAAGCGAG
OAS2	NM_016817.2	481-580	T023	NM_016817.2:480_T023	CTGGATGGTGAACCCATCAAGGGACTTCTGGATCTCGAAATTGTTTTTCACCACGCGATGACGTTCGTCAAGAGTCGCATAATCT
OAS3	NM_006187.2	4981-5080	T024	NM_006187.2:4980_T024	AGGTAAGTTTTAACAGTGGTTTGTGGAGAGTCAGGCTGTCTAAGGCACTCCATTTGGAATGATGTGTACTGGGAATAAGACGACG
OASL	NM_198213.1	261-360	T025	NM_198213.1:260_T025	TGTGATGCTTGGCTGCCTCCTGGAAGCTGTGGAAACAGCTCAGAAACGCACAAGAATCCCTGCTAGCTGAAGGAGGGTCAAAC
PLSCR1	NM_021105.2	1301-1400	T026	NM_021105.2:1300_T026	ATCCACTACCACACTCCTGATTTTTGTTCCTGGCTGCCAGCTTGACGTAGATTGCTATCAGGTTACGATGACTGC
RSAD2	NM_080657.4	1038-1137	T027	NM_080657.4:1037_T027	CCGTCCCTTTCTACAGTTCAGAAAGCGCATATATTCATCCAGAATAAGGTCTTACAGATCGTGTGCTCATGACTTCCACAGACGT
RTP4	NM_022147.2	476-575	T028	NM_022147.2:475_T028	CACAATTGGCTGTGTCATGGGATCCTTCCAGGGACACTTCCAGGATTACTCTTGGAGGAGTTGATAGTGGTAAAACAACATTAGC
SAMD9	NM_017654.2	6526-6625	T029	NM_017654.2:6525_T029	ACTCAGGACTAATCTTGTTTCGGTTCTACTTCCCTTTCACCACCCCCACACCTACGTATATATCCAAGTGGTTATGTCCGACGGC

SOCS1	NM_003745.1	1026-1125	T030	NM_003745.1:1025_T030	ATGAAGAGGTAGGAGGTGCGAGTTCAGGTCCTGGCCAGCAAGAAGGAGTATGGAACTTATAGCAAGAGAG
USP18	NM_017414.3	1459-1558	T031	NM_017414.3:1458_T031	GGAAATGGAAAATGACAGCGTGTCAATCTCTGAAGGTTTTGGGCATTTCCCACCCCTCCAAACGCATTCTTATTGGCAAATGGAA
ALAS1	NM_000688.4	396-495	T032	NM_000688.4:395_T032	CATCTTGGGGCAGTTTTGGGCATAGAACAACAGAGATTTGCCTGCTTTCTCCCGAAGCAATACTGTCGTCACTCTGTATGTCCGT
HPRT1	NM_000194.1	241-340	T033	NM_000194.1:240_T033	TGAGCACACAGAGGGCTACAATGTGATGGCCTCCCATCTCCTTCATCACACCGGGAATCGGCATTTCGCATTCTTAGGATCTAAA
TBP	NM_001172085.1	588-687	T034	NM_001172085.1:587_T034	GCACGAAGTGCAATGGTCTTTAGGTCAAGTTTACAACCAAGATTCACTGTCCGATCTTCATAACGGACAAACTGAACGGGCCATT
TUBB	NM_178014.2	1956-2055	T035	NM_178014.2:1955_T035	ACTGCTGACACCTCCCTTGAAGCTGAGATGGGAAATGGACATACTTAGAACGCTATGCAGACGAGCTGGCAGAGGAGAGAAATCA
SIGLEC1	NM_023068.3	5166-5265	T036	NM_023068.3:5165_T036	CAACACTGCCTCATTCACATTCATAGGCTGGAGTCATCACAGATTCTGCATTCGCAACCATGTGAAGTAATGTGAGCGTACTT

Supplemental Table 11. Nucleotide sequences for probe B

Gene Name	Accession	Position	Tag	Name	Sequence
BCL2L1	NM_001191.2	261-360	T001	NM_001191.2:260_ProbeB	CGAAAGCCATGACCTCCGATCACTCAATAGGGATGGGCTCAACCAGTCCATTGTCCAAAACACCTGCTCACTCAC
CD274	NM_014143.3	1246-1345	T002	NM_014143.3:1245_ProbeB	CGAAAGCCATGACCTCCGATCACTCGAACTGACCCTCAAATTAGGGATTCTCAACCCGTCTTCCTAGGAT
CXCL10	NM_001565.1	41-140	T003	NM_001565.1:40_ProbeB	CGAAAGCCATGACCTCCGATCACTCGAGAGAGGTACTCCTTGAATGCCACTTAGAGTCAGAAAGATAAGGCAGCA
DDX60	NM_017631.5	661-760	T004	NM_017631.5:660_ProbeB	CGAAAGCCATGACCTCCGATCACTCCTGTGTTTGTAGATCGTTCAGGCCTTCGTCTGCAACTATCAGGAAATATG
GBP1	NM_002053.1	2111-2210	T005	NM_002053.1:2110_ProbeB	CGAAAGCCATGACCTCCGATCACTCTGGGACATTGTAGACTTGGCCAGACCAATGCCCAAATATTTCCCAAGACT
HERC5	NM_016323.2	3092-3191	T006	NM_016323.2:3091_ProbeB	CGAAAGCCATGACCTCCGATCACTCAGCGCTTCTTCAACTGTTTCCATTGTAGAATATTTAGGGAGGAAGAGGAC
HERC6	NM_001165136.1	2076-2175	T007	NM_001165136.1:2075_ProbeB	CGAAAGCCATGACCTCCGATCACTCTACTTTGCAGAAGTCAGTAGCTTCAGCTTGACTTAATTGACGCAGAGCAT
IDO1	NM_002164.3	51-150	T008	NM_002164.3:50_ProbeB	CGAAAGCCATGACCTCCGATCACTCACTGATTGTCCAGGAGTTTTCCATAGCGTGTGCCATTCTTGTAGTCTGCT
IFI27	NM_005532.3	391-490	T009	NM_005532.3:390_ProbeB	CGAAAGCCATGACCTCCGATCACTCGGAGCTAGTAGAACCTCGCAATGACAGCCGCAATGGCAGACCCAATG
IFI44	NM_006417.4	751-850	T010	NM_006417.4:750_ProbeB	CGAAAGCCATGACCTCCGATCACTCGCCATCTTTCCCGTCTCTAATAGAGTATGTCCTATACTTCTCAGATATCC
IFI44L	NM_006820.2	941-1040	T011	NM_006820.2:940_ProbeB	CGAAAGCCATGACCTCCGATCACTCCATACAACCTTTTAAGATGTGGGGAATGTCATCCATGCACAGTCCTGCTC
IFI6	NM_002038.3	181-280	T012	NM_002038.3:180_ProbeB	CGAAAGCCATGACCTCCGATCACTCCGACGGCCATGAAGGTCAGGGCCTTCCAGAACCCG
IFIT1	NM_001548.3	1441-1540	T013	NM_001548.3:1440_ProbeB	CGAAAGCCATGACCTCCGATCACTCCAGGGCCCGCTCATAGTACTCCAGGGCTTCATTCATATTTCCTTCCAATT
IFIT2	NM_001547.4	1996-2095	T014	NM_001547.4:1995_ProbeB	CGAAAGCCATGACCTCCGATCACTCGCGATGGGGGATACTGAGCCCTATTTAGACTTTGGTCCGCCAGCTTTTGG
IFIT3	NM_001031683.2	1471-1570	T015	NM_001031683.2:1470_ProbeB	CGAAAGCCATGACCTCCGATCACTCCTGGCCCATTTCCTCACTACCATCCTCAAGCTCAGAT
IFIT5	NM_012420.2	3588-3687	T016	NM_012420.2:3587_ProbeB	CGAAAGCCATGACCTCCGATCACTCCTACTCAAAATGTAATTAGAACAGCCACAGCATCCTCACCTAGGAGGAGC
IFNA2	NM_000605.3	12-111	T017	NM_000605.3:11_ProbeB	CGAAAGCCATGACCTCCGATCACTCTGAGCACCAGGAGGGCCACCAGTAAAGCAAAGGTCAAGGCCATTGTAGAT
ISG15	NM_005101.3	306-405	T018	NM_005101.3:305_ProbeB	CGAAAGCCATGACCTCCGATCACTCTGCTGCTGCGGCCCTTGTTATTCCTCACCAGGATG
LAMP3	NM_014398.3	879-978	T019	NM_014398.3:878_ProbeB	CGAAAGCCATGACCTCCGATCACTCCTTGTCTTGAACAATCAGCTGTATCCCCATCTCTGCTTTTATACAGAGT
LY6E	NM_002346.2	381-480	T020	NM_002346.2:380_ProbeB	CGAAAGCCATGACCTCCGATCACTCCCATCGGCCGCACTGAAATTGCACAGAAAGCTCTGGCAGCAGC
MX1	NM_002462.2	1486-1585	T021	NM_002462.2:1485_ProbeB	CGAAAGCCATGACCTCCGATCACTCATTTGTGGAACTCGTGTCGGAGTCTGGTAAACAGCCGAATGTCTTCCTCC
OAS1	NM_016816.2	6-105	T022	NM_016816.2:5_ProbeB	CGAAAGCCATGACCTCCGATCACTCGACAGGAGAGAGAGAGTGGCAACAGAACTGCCTCCCAGACCTGTTTGCTTTT
OAS2	NM_016817.2	481-580	T023	NM_016817.2:480_ProbeB	CGAAAGCCATGACCTCCGATCACTCTTGAAGGCGGCCAGCACCTCGAAAGAGATTCTCTGATTTTTTGTGAACAC
OAS3	NM_006187.2	4981-5080	T024	NM_006187.2:4980_ProbeB	CGAAAGCCATGACCTCCGATCACTCCAATAATGAGGGAAGGCTCTTCCCATCCCATTCAATCTAGCATTCCTAGC
OASL	NM_198213.1	261-360	T025	NM_198213.1:260_ProbeB	CGAAAGCCATGACCTCCGATCACTCCAGGTCCTGGCTTTGCCACATGGTTTTCCATATCAGCCTCAGAACATCTT
PLSCR1	NM_021105.2	1301-1400	T026	NM_021105.2:1300_ProbeB	CGAAAGCCATGACCTCCGATCACTCTAGTCTCAATCAATATACAGACTTCAGATTTCCTGAGGAGACTTTCACTA
RSAD2	NM_080657.4	1038-1137	T027	NM_080657.4:1037_ProbeB	CGAAAGCCATGACCTCCGATCACTCTTTATAGCTTCTTCTACACCAACATCCAGGATGGACTTGGAAGGGTCCTT
RTP4	NM_022147.2	476-575	T028	NM_022147.2:475_ProbeB	CGAAAGCCATGACCTCCGATCACTCCATGCAGCTTTTTAAGCCCTGTCCACAGATGCCCAAAGTGCATGCCT

SAMD9	NM_017654.2	6526-6625	T029	NM_017654.2:6525_ProbeB	CGAAAGCCATGACCTCCGATCACTCAGGGAGAAAAGTGTGCTGAATTCCATGTATCCAGCTTGCAGCCATTGTTA
SOCS1	NM_003745.1	1026-1125	T030	NM_003745.1:1025_ProbeB	CGAAAGCCATGACCTCCGATCACTCGACCCTCCCCCAACCCCTGGTTTGTGCAAAGATACTGGGTATATGTAAAC
USP18	NM_017414.3	1459-1558	T031	NM_017414.3:1458_ProbeB	CGAAAGCCATGACCTCCGATCACTCTGCTTCTCCTCATTGCAAAATCTCTTAGAAGACTCCGTAGATCCAGGAAC
ALAS1	NM_000688.4	396-495	T032	NM_000688.4:395_ProbeB	CGAAAGCCATGACCTCCGATCACTCAATGCCCGAGGGGCTGGCTTGGCCCCAACTTCCAT
HPRT1	NM_000194.1	241-340	T033	NM_000194.1:240_ProbeB	CGAAAGCCATGACCTCCGATCACTCCAGTGCTTTGATGTAATCCAGCAGGTCAGCAAAGAATTTATAGCCCCCCT
TBP	NM_001172085.1	588-687	T034	NM_001172085.1:587_ProbeB	CGAAAGCCATGACCTCCGATCACTCTCCTCATGATTACCGCAGCAAACCGCTTGGGATTATATTCGGCGTTTCGG
TUBB	NM_178014.2	1956-2055	T035	NM_178014.2:1955_ProbeB	CGAAAGCCATGACCTCCGATCACTCACAGACTCCTCCAGAGTAGAGCTTGGAGGGAGATTGAAAGTGGAGATAAT
SIGLEC1	NM_023068.3	5166-5265	T036	NM_023068.3:5165_ProbeB	CGAAAGCCATGACCTCCGATCACTCCATAAAAAGTCAGATGTCACAGAGCTGTTTTCGTAGAGGCGGGCAGGACT

Supplemental Table 12. Synthetic DNA oligonucleotides used as a standard

Gene Name	Sequence
BCL2L1	ATC TTG GCT TTG GAT CTT AGA AGA GAA TCA CTA ACC AGA GAC GAG ACT CAG TGA GTG AGC AGG TGT TTT GGA CAA TGG ACT GGT TGA GCC CAT CCC TAT T
CD274	GGA TAC TTC TGA ACA AGG AGC CTC CAA GCA AAT CAT CCA TTG CTC ATC CTA GGA AGA CGG GTT GAG AAT CCC TAA TTT GAG GGT CAG TTC CTG CAG AAG T
CXCL10	GCA GAG GAA CCT CCA GTC TCA GCA CCA TGA ATC AAA CTG CGA TTC TGA TTT GCT GCC TTA TCT TTC TGA CTC TAA GTG GCA TTC AAG GAG TAC CTC TCT C
DDX60	TTC GAG ATG CTT ATC AAA AGA GTG GGG AAG TTT CTT GGA AGA GAG TTA CCC ATA TTT CCT GAT AGT TGC AGA CGA AGG CCT GAA CGA TCT ACA AAC ACA G
GBP1	CCA GAT GAC CAG CAG TAG ACA AAT GGA TAC TGA GCA GAG TCT TAG GTA AAA GTC TTG GGA AAT ATT TGG GCA TTG GTC TGG CCA AGT CTA CAA TGT CCC A
HERC5	CTG AAA GTT GGA ATG AAA GAG ACC CTA TAA GAG CAC TGA CAT GTT TCA GTG TCC TCT TCC TCC CTA AAT ATT CTA CAA TGG AAA CAG TTG AAG AAG CGC T
HERC6	TCC ATC ACC CAG ATT TAT ACT TAG AGT CAG ACG AAG TCG CCT GGT TAA AGA TGC TCT GCG TCA ATT AAG TCA AGC TGA AGC TAC TGA CTT CTG CAA AGT A
IDO1	CTA TTA TAA GAT GCT CTG AAA ACT CTT CAG ACA CTG AGG GGC ACC AGA GGA GCA GAC TAC AAG AAT GGC ACA CGC TAT GGA AAA CTC CTG GAC AAT CAG T
IFI27	TCA CTG GGA GCA ACT GGA CTC TCC GGA TTG ACC AAG TTC ATC CTG GGC TCC ATT GGG TCT GCC ATT GCG GCT GTC ATT GCG AGG TTC TAC TAG CTC CCT G
IFI44	TTT CCA AGG GCA TGT AAC GCA TCA GGC TTT GGT GGG CAC TAA TAC AAC TGG GAT ATC TGA GAA GTA TAG GAC ATA CTC TAT TAG AGA CGG GAA AGA TGG C
IFI44L	ATC TCT GCC ATT TAT GTT GTG TGA CAC TAT GGG GCT AGA TGG GGC AGA AGG AGC AGG ACT GTG CAT GGA TGA CAT TCC CCA CAT CTT AAA AGG TTG TAT G
IFI6	GGG GTG GAG GCA GGT AAG AAA AAG TGC TCG GAG AGC TCG GAC AGC GGC TCC GGG TTC TGG AAG GCC CTG ACC TTC ATG GCC GTC GGA GGA GGA CTC GCA G
IFIT1	GAG AAA GGC ATT AGA TCT GGA AAG CTT GAG CCT CCT TGG GTT CGT CTA CAA ATT GGA AGG AAA TAT GAA TGA AGC CCT GGA GTA CTA TGA GCG GGC CCT G
IFIT2	TGC ATC CCA TAG AGG TTA GTC CTG CAT AGC CAG TAA TGT GCT AAG TTC ATC CAA AAG CTG GCG GAC CAA AGT CTA AAT AGG GCT CAG TAT CCC CCA TCG C
IFIT3	CGC CTG CTA AGG GAT GCC CCT TCA GGC ATA GGC AGT ATT TTC CTG TCA GCA TCT GAG CTT GAG GAT GGT AGT GAG GAA ATG GGC CAG GGC GCA GTC AGC T
IFIT5	GGG CTG TGT TCA TAC ACA GAA GGG GCC TGA GAT TTC TGC ACT TTA AAC AAG CTC CTC CTA GGT GAG GAT GCT GTG GCT GTT CTA ATT ACA TTT TGA GTA G
IFNA2	GCC TAA GGT TTA GGC TCA CCC ATT TCA ACC AGT CTA GCA GCA TCT GCA ACA TCT ACA ATG GCC TTG ACC TTT GCT TTA CTG GTG GCC CTC CTG GTG CTC A
ISG15	CCC GGC AGC ACG GTC CTG CTG GTG GTG GAC AAA TGC GAC GAA CCT CTG AGC ATC CTG GTG AGG AAT AAC AAG GGC CGC AGC AGC ACC TAC GAG GTA CGG C
LAMP3	CAG CCA TCG TCA GTC AAG ACT GGA ATT TAT CAG GTT CTA AAC GGA AGC AGA CTC TGT ATA AAA GCA GAG ATG GGG ATA CAG CTG ATT GTT CAA GAC AAG G
LY6E	CTG CCC CAT CCC AGA AGG CGT CAA TGT TGG TGT GGC TTC CAT GGG CAT CAG CTG CTG CCA GAG CTT TCT GTG CAA TTT CAG TGC GGC CGA TGG CGG GCT G
MX1	GCC TTT AAT CAG GAC ATC ACT GCT CTC ATG CAA GGA GAG GAA ACT GTA GGG GAG GAA GAC ATT CGG CTG TTT ACC AGA CTC CGA CAC GAG TTC CAC AAA T
OAS1	TCT GAG GAA ACG AAA CCA ACA GCA GTC CAA GCT CAG TCA GCA GAA GAG ATA AAA GCA AAC AGG TCT GGG AGG CAG TTC TGT TGC CAC TCT CTC TCC TGT C
OAS2	TGA AAA ACA ATT TCG AGA TCC AGA AGT CCC TTG ATG GGT TCA CCA TCC AGG TGT TCA CAA AAA ATC AGA GAA TCT CTT TCG AGG TGC TGG CCG CCT TCA A
OAS3	GAG TGC CTT AGA CAG CCT GAC TCT CCA CAA ACC ACT GTT AAA ACT TAC CTG CTA GGA ATG CTA GAT TGA ATG GGA TGG GAA GAG CCT TCC CTC ATT ATT G
OASL	GGC GTT TCT GAG CTG TTT CCA CAG CTT CCA GGA GGC AGC CAA GCA TCA CAA AGA TGT TCT GAG GCT GAT ATG GAA AAC CAT GTG GCA AAG CCA GGA CCT G
PLSCR1	TTT GAA AGC ACT GGC AGC CAG GAA CAA AAA TCA GGA GTG TGG TAG TGG ATT AGT GAA AGT CTC CTC AGG AAA TCT GAA GTC TGT ATA TTG ATT GAG ACT A
RSAD2	ACC TTA TTC TGG ATG AAT ATA TGC GCT TTC TGA ACT GTA GAA AGG GAC GGA AGG ACC CTT CCA AGT CCA TCC TGG ATG TTG GTG TAG AAG AAG CTA TAA A
RTP4	AGT AAT CCT GGA AGT GTC CCT GGA AGG ATC CCA TGA CAC AGC CAA TTG TGA GGC ATG CAC TTT GGG CAT CTG TGG ACA GGG CTT AAA AAG CTG CAT GAC A
SAMD9	TGT GGG GGT GGT GAA AGG GAA GTA GAA CCG AAA CAA GAT TAG TCC TGA GTT AAC AAT GGC TGC AAG CTG GAT ACA TGG AAT TCA GCA CAC TTT TCT CCC T

SIGLEC1	GCC AGA ATC TGT GAT GAC TCC AGC CTA TGA ATG TGA ATG AGG CAG TGT TGA GTC CTG CCC GCC TCT ACG AAA ACA GCT CTG TGA CAT CTG ACT TTT TAT G
SOCS1	TTA ACT GTA TCT GGA GCC AGG ACC TGA ACT CGC ACC TCC TAC CTC TTC ATG TTT ACA TAT ACC CAG TAT CTT TGC ACA AAC CAG GGG TTG GGG GAG GGT C
USP18	GGA AAT GCC CAA AAC CTT CAG AGA TTG ACA CGC TGT CAT TTT CCA TTT CCG TTC CTG GAT CTA CGG AGT CTT CTA AGA GAT TTT GCA ATG AGG AGA AGC A
ALAS1	AGA AAG CAG GCA AAT CTC TGT TGT TCT ATG CCC AAA ACT GCC CCA AGA TGA TGG AAG TTG GGG CCA AGC CAG CCC CTC GGG CAT TGT CCA CTG CAG CAG T
HPRT1	TGT GAT GAA GGA GAT GGG AGG CCA TCA CAT TGT AGC CCT CTG TGT GCT CAA GGG GGG CTA TAA ATT CTT TGC TGA CCT GCT GGA TTA CAT CAA AGC ACT G
TBP	ACA GTG AAT CTT GGT TGT AAA CTT GAC CTA AAG ACC ATT GCA CTT CGT GCC CGA AAC GCC GAA TAT AAT CCC AAG CGG TTT GCT GCG GTA ATC ATG AGG A
TUBB	TTC TAA GTA TGT CCA TTT CCC ATC TCA GCT TCA AGG GAG GTG TCA GCA GTA TTA TCT CCA CTT TCA ATC TCC CTC CAA GCT CTA CTC TGG AGG AGT CTG T

Supplemental Table 13. Overview of Criteria used to Obtain Optimal Values on ROC Curves- Prevalence

**Cut-point**	**Criteria***
**Sensitivity = Specificity**	The point on the ROC curve at which the sensitivity = specificity is the point on the ROC curve at which the product of sensitivity and specificity is maximized. It is also the point of intersection between the ROC curve and the line that connects (0,1) and (1,0).
**Maximum Youden's Index**	The point on the ROC curve at which Youden's index (sensitivity + specificity – 1) is maximized. This is the point on the ROC curve at which the sum of sensitivity and specificity is maximized. Since sensitivity = true-positive (TP) rate and 1 – specificity = false-positive (FP) rate, this is also the point at which the difference between the TP and FP rate is maximized. In addition, in the ROC plot of sensitivity versus 1-specificity, the 45-degree line represents an uninformative test. The vertical distance between any point on the ROC curve and a point on the 45-degree line is the distance between (1 – specificity, 1 – specificity) and (1-specificity, sensitivity) = (sensitivity + specificity – 1)^2. Therefore, the point on the ROC at which Youden's index is maximized is also the point that maximizes the vertical distance between the ROC curve and the 45-degree line. Since the points on the 45-degree line satisfy TP = FP, the 45-degree line is the ROC curve for an uninformative test. The maximum value of the Youden's index is therefore the point on the ROC curve that has the greatest vertical distance from the ROC curve for an uninformative test.
**Minimum Distance from [FP=0,TP=1] (Upper-Left Corner of Unit Square)**	This point on the ROC curve is the point at which the distance from (0,1) (the point corresponding to FP = 0 and TP = 1) is minimized.
**Maximum Weighted NNM (C=2, prevalence = 5%,10%,15%)**	This is the cut-point at which the Weighted Number Needed to Misdiagnose (NNM) is maximized, where the NNM represents the number of samples that need to be tested in order for one sample to be misdiagnosed. The probability of misdiagnosis = P(+ test result | sample is non-AGS) x P(sample is non-AGS) + P(- test result | sample is AGS) x P(sample is AGS) = (1 – specificity) x (1 – prevalence) + (1 – sensitivity) x prevalence. If the probability of misdiagnosis is 0.5, the number of samples that need to be tested in order for one sample to be misdiagnosed is 2. We consider the Weighted Number Needed to Misdiagnose (proposed by Habibzadeh) because we are more concerned about the harm due to a false negative (FN) than the harm due to

a false positive (FP). We let C = the harm due to FN divided by the harm due to FP. We assume C = 2 and obtain the cut-point at which the Weighted Number Needed to Misdiagnose is maximized, where the Weighted Number to Misdiagnose = 1/[C x FN + FP] = 1/[(1 – specificity) x (1 – prevalence) + C x (1 – sensitivity) x prevalence].

1. Habibzadeh F, Habibzadeh P, and Yadollahie M. On determining the most appropriate test cut-off value: the case of tests with continuous results. *Biochem Med (Zagreb).* 2016;26(3):297-307.
2. Crow YJ, Chase DS, Lowenstein Schmidt J, Szynkiewicz M, Forte GM, Gornall HL, et al. Characterization of human disease phenotypes associated with mutations in TREX1, RNASEH2A, RNASEH2B, RNASEH2C, SAMHD1, ADAR, and IFIH1. *Am J Med Genet A.* 2015;167a(2):296-312.
3. Rice GI, Forte GM, Szynkiewicz M, Chase DS, Aeby A, Abdel-Hamid MS, et al. Assessment of interferon-related biomarkers in Aicardi-Goutieres syndrome associated with mutations in TREX1, RNASEH2A, RNASEH2B, RNASEH2C, SAMHD1, and ADAR: a case-control study. *The Lancet Neurology.* 2013;12(12):1159-69.
4. Rice GI, Melki I, Fremond ML, Briggs TA, Rodero MP, Kitabayashi N, et al. Assessment of Type I Interferon Signaling in Pediatric Inflammatory Disease. *J Clin Immunol.* 2017;37(2):123-32.
